# RNA Helicase DDX21 Controls CD4^+^ T Cell Proliferation and Promotes Inflammatory Bowel Disease via Translational Control

**DOI:** 10.1002/advs.202516653

**Published:** 2026-05-29

**Authors:** Yujuan Zhang, Chen Kan, Xinhui Yang, Yajuan Hao, Xuemin Cai, Mei Yang, Qianqian Zhang, Zhengting Wang, Ning Jiang, Lei Wang, Hua‐Bing Li, Jing Zhou

**Affiliations:** ^1^ Department of Geriatrics Medical Center on Aging of Ruijin Hospital Shanghai Jiao Tong University School of Medicine Shanghai China; ^2^ Center For Immune‐Related Diseases At Shanghai Institute of Immunology Ruijin Hospital Shanghai Jiao Tong University School of Medicine Shanghai China; ^3^ Shanghai Jiao Tong University School of Medicine‐Yale Institute For Immune Metabolism Shanghai Jiao Tong University School of Medicine Shanghai China; ^4^ School of Biological Science The University of Manchester Manchester UK; ^5^ Department of Gastroenterology Ruijin Hospital Shanghai Jiao Tong University School of Medicine Shanghai China; ^6^ Department of Urology Shanghai Tenth People's Hospital Tongji University Shanghai China; ^7^ Urologic Cancer Institute School of Medicine Tongji University Shanghai China; ^8^ Institute For Immunology and Pathogenesis Chongqing Medical University Chongqing China; ^9^ Institute of Frontier Technologies and Interdisciplinary Research Chongqing Medical University Chongqing China; ^10^ Department of Pathology The First Affiliated Hospital of Chongqing Medical University Chongqing China; ^11^ Key Laboratory of Cell Differentiation and Apoptosis of Chinese Ministry of Education Shanghai Jiao Tong University School of Medicine Shanghai China

**Keywords:** inflammatory bowel disease, mRNA translation, RNA helicase, T cell homeostasis

## Abstract

Inflammatory bowel disease (IBD) is characterized by dysregulated T cell responses. RNA helicases, including DExD‐box helicase 21 (DDX21), are pivotal in RNA metabolism, but their role in T cell‐mediated pathology during IBD remains unclear. Here, we demonstrate that *DDX21* expression in CD4^+^ T cells correlates with cell cycle and translation pathways in IBD‐affected tissues. Conditional deletion of *Ddx21* in mouse T cells disrupts T cell homeostasis and impairs cell proliferation. Consequently, *Ddx21*–deficient CD4^+^ T cells exhibit resistance to inducing adoptive transfer colitis, with recipients showing reduced T cell infiltration compared to wild‐type (WT) counterparts. Mechanistically, DDX21 ensures ribosome biogenesis after T cell activation and facilitates mRNA translation of Transcription factor dp‐1 (TFDP1), a transcription factor critical for cell cycle progression. Given this dependence on ribosome biogenesis, pharmacological targeting of this pathway via KU55933—an inhibitor of ribosome synthesis‐related signaling—recapitulated the protective effects of DDX21 loss: KU55933 alleviated dextran sulfate sodium (DSS)‐induced colitis in mice and attenuated pathogenic CD4^+^ T cell expansion. Our findings establish DDX21 as a key regulator of T cell proliferation and highlight its potential as a therapeutic target for IBD and other autoimmune disorders.

## Graphical abstract

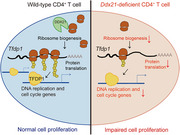

## 1 Introduction

Inflammatory bowel disease (IBD), comprising Crohn's disease and ulcerative colitis (UC), affects approximately 7 million individuals globally [1]. Patients with IBD may have an increased risk of depression and anxiety compared with the general population, leading to a decline in quality of life and treatment challenges [2]. An imbalanced T cell immune response is implicated in IBD development [3, 4, 5], underscoring the need for deeper research into the molecular mechanisms governing T cell behavior during disease progression.

Naïve T cells are initially dormant but become rapidly activated upon antigen receptor and co‐signaling pathway stimulation [6, 7]. This activation triggers cell cycle entry and clonal expansion. Subsequently, a series of coordinated transcriptional and post‐transcriptional events drives their differentiation into effector subsets [8, 9, 10, 11, 12]. Notably, within 24 hours of activation, T cells increase ribosomal output over 13‐fold to support accelerated protein synthesis for proliferation [13]. Transcriptomic analyses indicate that genes upregulated in expanded T cells predominantly encode ribosomal and nucleosomal components [14, 15], highlighting ribosomes’ pivotal role in transitioning T cells from quiescence. Given that translation kinetics dictate T cell proliferation and function [16], it is essential to elucidate the molecular regulators associated with ribosomes.

RNA‐binding proteins (RBPs) serve as crucial regulators of RNA metabolism, overseeing various processes ranging from pre‐mRNA processing to translation and degradation [17]. DExD‐box helicase 21 (DDX21), a multifunctional DEAD‐box helicase, is crucial for ribosome biogenesis [18, 19]. It regulates RNA polymerase I transcription [18] and facilitates small nucleolar RNA recruitment to pre‐ribosomal complexes [20]. DDX21 also participates in epigenetic silencing [21] and improves disease‐free survival through the regulation of c‐Jun and rRNA biogenesis [22]. Despite emerging roles of this RNA helicase, its involvement in the translational control of T cells has not been thoroughly investigated.

In this study, we reveal a correlation between *DDX21* expression in CD4^+^ T cells and cell cycle/translation pathways within IBD‐impacted tissues. When *Ddx21* is conditionally deleted in mouse T cells, T cell homeostasis is compromised, and cell proliferation is hindered. As a result, *Ddx21*–deficient CD4^+^ T cells display resistance to inducing adoptive transfer colitis. Compared with recipients of wild‐type (WT) CD4^+^ T cells, those receiving *Ddx21*–deficient CD4^+^ T cells show notably reduced T cell infiltration and cytokine production. Mechanistically, DDX21 is crucial for ensuring ribosome biogenesis following T cell activation and supports the mRNA translation of Transcription factor dp‐1 (TFDP1), a transcription factor vital for cell cycle progression. To therapeutically exploit this mechanism, targeting this ribosomal pathway with KU55933—an inhibitor of ribosome biogenesis signaling—mimicked *Ddx21* deficiency, alleviating dextran sulfate sodium (DSS)‐induced colitis and attenuating pathogenic CD4^+^ T cell expansion in mice. Overall, our study identifies DDX21 as a pivotal regulator of T cell expansion, suggesting its potential as a therapeutic target for IBD and other autoimmune diseases.

## 2 Results

### 2.1 *DDX21* Expression Correlates With T Cell Proliferation in Human IBD

**FIGURE 1:**
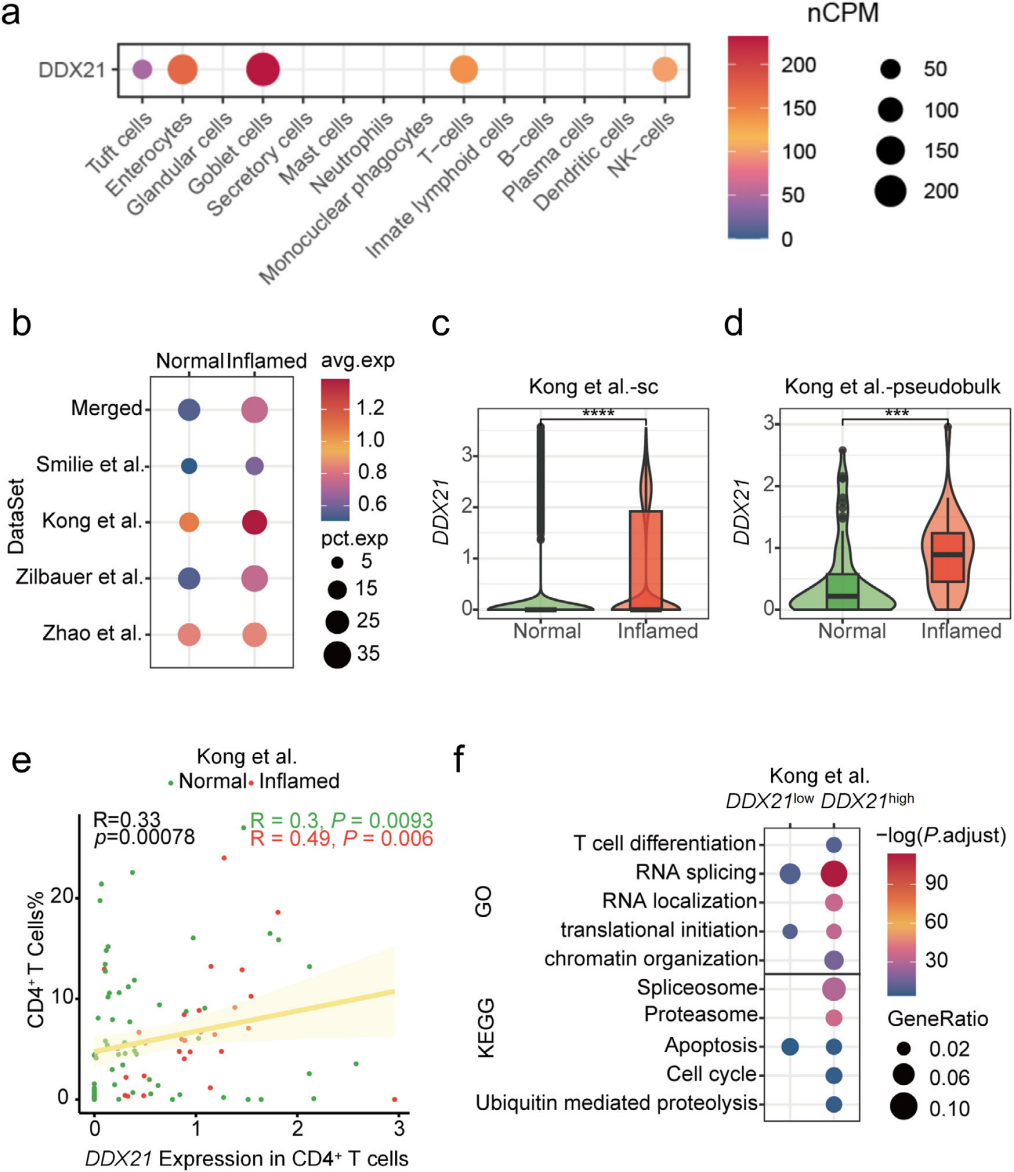
*DDX21* expression in CD4^+^ T cells correlates with cell proliferation signaling in inflammatory bowel disease. (a) Dot plots display the expression of DDX21 (represented by dot size and dot color) in cells from the normal colon in The Human Protein Atlas (https://www.proteinatlas.org/). (b) Dot plots display the average expression (indicated by dot color) and the percentage of cells expressing *DDX21* (represented by dot size) in CD4^+^ T cells from normal and inflamed tissues across various inflammatory bowel disease (IBD) datasets (Merged: combining the used datasets; Smillie et al. (Single Cell Portal: SCP25922 [23]); Kong et al. (Single Cell Portal: SCP188423 [24]); Zilbauer et al. (Biostudies: E‐MTAB‐890124 [25]); and Zhao et al. (GEO accession: GSE24208725 [26])). (c,d) ‌Violin plots illustrate the comparison of *DDX21* expression levels in CD4^+^ T cells between normal and inflamed tissues, utilizing data from Kong et al. (c) presents single‐cell level analysis, while (d) depicts the pseudobulk‐level analysis. The Wilcoxon rank‐sum test was employed to assess statistical significance, with ^***^ indicating *p* < 0.001 and ^****^ representing *p* < 0.0001. (e) Scatter plot with a Spearman correlation coefficient indicates the relationship between *DDX21* expression in CD4^+^ T cells and the proportion of CD4^+^ T cells, utilizing data from Kong et al. (f) Gene Ontology (GO) and Kyoto Encyclopedia of Genes and Genomes (KEGG) enrichment analysis results for *DDX21* ^high/low^ CD4^+^ T cells in Kong et al.’s dataset are shown.

To analyze DDX21 expression across different colonic cell types, we examined data from The Human Protein Atlas (https://www.proteinatlas.org/). This analysis showed that DDX21 exhibited the highest expression in T cells relative to other major immune cell populations in the colon (Figure 1a). To further explore the role of DDX21 in regulating T cell activity during IBD, we analyzed previously published single‐cell transcriptomes from both inflamed and non‐inflamed colonic tissues in IBD patients [23, 24, 25, 26]. Our analysis revealed that *DDX21* expression in CD4^+^ T cells was significantly elevated in inflamed tissues compared to normal tissues (Figure 1b–d). Specifically, higher *DDX21* expression correlated with an increased proportion of CD4^+^ T cells (R = 0.33, *p* = 0.00078; Figure 1e), with this correlation being even stronger under inflammatory conditions (R = 0.49, *p* = 0.006; Figure 1e). Further enrichment analysis using Gene Ontology (GO) and the Kyoto Encyclopedia of Genes and Genomes (KEGG) on CD4^+^ T cells with high vs. low *DDX21* expression, based on data from Kong et al. [24], revealed that several cell proliferation‐related pathways were enriched in *DDX21* ^high^ CD4^+^ T cells, including T cell differentiation, RNA splicing, RNA localization, translational initiation, chromatin organization, spliceosome, proteasome, apoptosis, cell cycle, and ubiquitin‐mediated proteolysis (Figure 1f). These findings suggest that *DDX21* expression in CD4^+^ T cells is linked to the regulation of proliferation in IBD.

### 2.2 DDX21 is Essential for Maintaining T Cell Homeostasis

Naïve T cell activation progresses through several defined stages: early signaling activation (0–6 hours), metabolic reprogramming (6–12 hours), pre‐cell cycle (12–24 hours), and proliferation (24–72 hours) [27, 28]. To systematically investigate the dynamic changes of DDX21 during T cell activation, we first analyzed published time‐course datasets, including proteomic [8] and RNA‐seq [29] data from T cells stimulated with anti‐CD3/CD28 antibodies. This analysis revealed a rapid upregulation of DDX21 upon activation (Figure S1a,b). To confirm these findings experimentally, we isolated naïve CD4^+^ T cells from WT mouse spleens and activated them in vitro with anti‐CD3/CD28 antibodies for 0, 6, 24, 48, and 72 hours. Western blot and real‐time PCR analysis demonstrated a significant induction of *Ddx21* following activation (Figure S1c,d). These findings suggest that DDX21 may have a regulatory role in T cell function.

**FIGURE 2:**
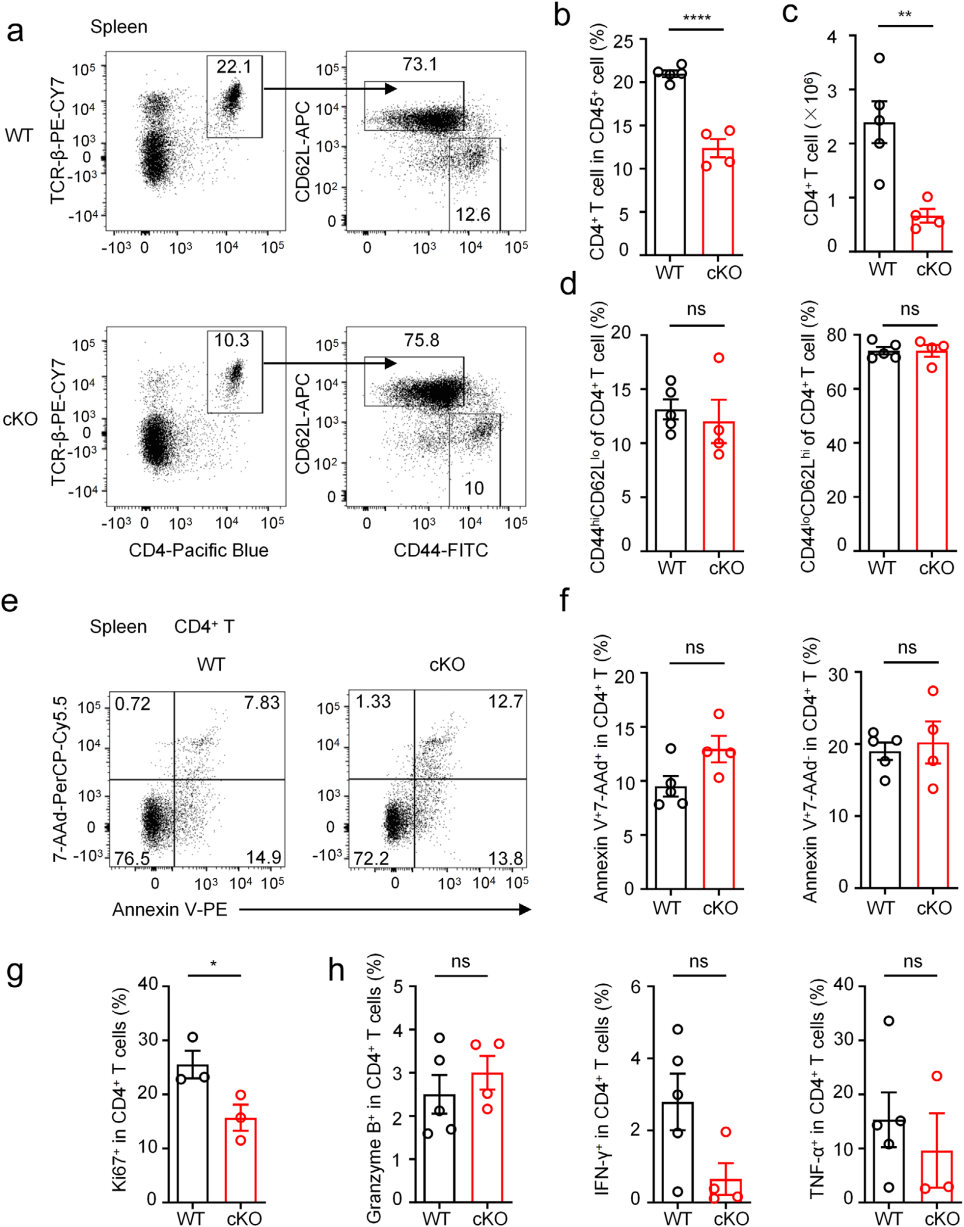
T cell‐specific *Ddx21* deletion disrupts peripheral CD4^+^ T cell homeostasis. (a) Representative dot plots show CD4^+^ T cell composition, as well as the expression of CD44 and CD62L on CD4^+^ T cells from *Ddx21*‐conditional knockout (cKO) and wild‐type (WT) littermate mice at a steady state in the spleen. (b,c) Ratios (b) and absolute numbers (c) of CD4^+^ T cells in the spleen are shown. (d) Ratios of CD44^lo^ CD62L^hi^ and CD44^hi^ CD62L^lo^ cells in splenic CD4^+^ T cells are shown. (e) Representative dot plots showing apoptosis in splenic CD4^+^ T cells. (f) Percentage of apoptotic CD4^+^ T cells in the spleen. (g) Percentage of Ki67^+^ CD4^+^ T cells in the spleen. (h) Percentage of IFN‐γ^+^, TNF‐α^+^, and Granzyme B^+^ cells among CD4^+^ T cells in the spleen of *Ddx21*‐cKO mice and WT littermates. Data represent one of three independent experiments and are shown as the means ± SEM, *n* = 3–5 biologically independent samples. ^*^ *p* < 0.05, ^**^ *p* < 0.01, ^****^ *p* < 0.0001. NS, not significant; results were analyzed by an unpaired *t*‐test.

To elucidate the function of DDX21 in T cells, we constructed *Ddx21* ^flox/flox^ mice using the CRISPR‐Cas9 method and crossed them with *Cd4* ^Cre^ mice to obtain *Ddx21* ^flox/flox^ *Cd4* ^Cre^ (*Ddx21*‐cKO) offspring (Figure S3a), in which DDX21 protein expression was specifically absent in CD4^+^ T cells (Figure S3b). To further explore the role of DDX21 in the maintenance of T cell homeostasis in vivo, we compared the development of T cells in the thymus and spleen between WT littermates and *Ddx21*‐cKO mice. We observed that the absence of DDX21 did not disrupt the late stages of T cell development in the thymus (Figure S3c–e) but led to a reduced proportion and number of both CD4^+^ T and CD8^+^ T cells in the spleen (Figure 2a–c and Figure S4a–c). Further analysis of the proportions of naïve and effector memory (EM) T cells in the spleen revealed that *Ddx21* knockout did not affect the activation status of CD4^+^ T cells (Figure 2a,d). However, it resulted in a decrease in the proportion of naïve CD8^+^ T cells and a corresponding increase in the proportion of EM CD8^+^ T cells (Figure S4a,d). Additionally, we assessed the proliferation and apoptosis of CD4^+^ and CD8^+^ T cells in the spleen of *Ddx21*‐cKO mice and compared them with those of WT mice. Our results indicated that *Ddx21*–deficient CD8^+^ T cells exhibited a higher rate of apoptosis compared to their WT counterparts (Figure S4e–g), while *Ddx21*–deficient CD4^+^ T cells exhibited decreased proliferation without affecting their apoptosis status (Figure 2e–g). Furthermore, we measured cytokine expression in CD4^+^ and CD8^+^ T cells in the spleen and found that only Granzyme B secretion was significantly higher in *Ddx21*–deficient CD8^+^ T cells than in WT cells (Figure S4h), without affecting cytokine production in CD4^+^ T cells (Figure 2h). Collectively, these results suggest that DDX21 promotes T cell homeostasis maintenance in peripheral immune organs.

### 2.3 DDX21 Deficiency Attenuates T Cell‐Driven Autoimmunity

**FIGURE 3:**
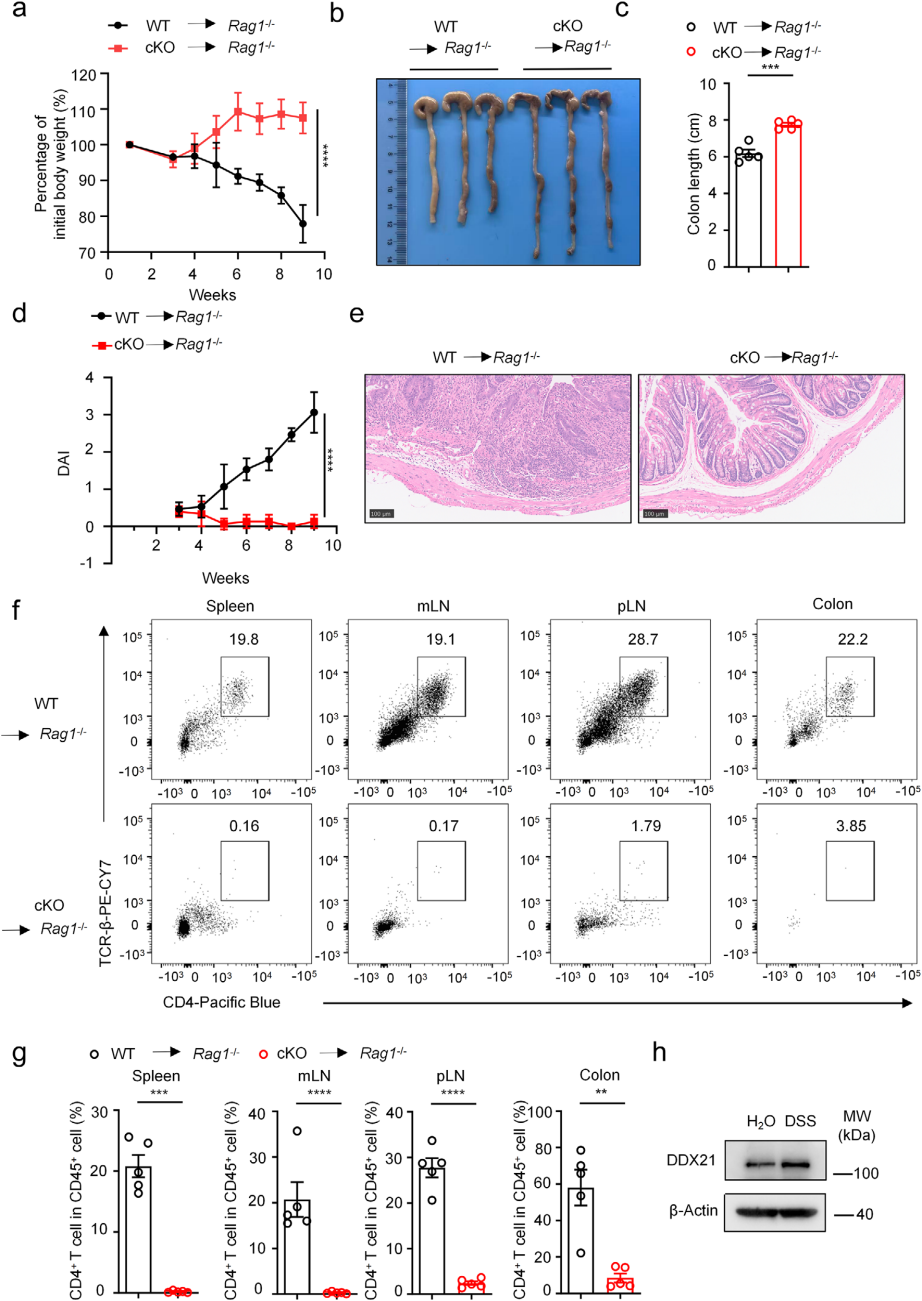
DDX21 deficiency attenuates T cell‐driven colitis. (a) Naïve CD4^+^ T cells (5 × 10^5^) were isolated from *Ddx21*‐cKO and WT mice and then transferred into *Rag1^−/−^* mice. The body weights of recipient mice were measured weekly and analyzed by two‐way analysis of variance (ANOVA) (*n =* 5). (b) The colons of mice from (a) were obtained 9 weeks after the adoptive transfer colitis model (*n =* 5). (c) The colon length of the recipient mice was measured from the caecum to the proximal rectum in each group (*n =* 5); results were analyzed by an unpaired *t*‐test. (d) The disease activity index (DAI) of recipient mice was measured weekly and analyzed by two‐way analysis of variance (ANOVA) (*n =* 5). (e) Representative images of hematoxylin and eosin (H&E) staining of the colons of *Rag1* ^−/−^ mice receiving WT or *Ddx21*‐cKO naive CD4^+^ T cells 9 weeks post‐transfer (*n*  = 5); scale bar = 100 µm. (f) Flow cytometric analysis of transferred CD4^+^ T cell population in the spleen, peripheral lymph node (pLN), mesenteric lymph node (mLN), and colon of the recipient mice 9 weeks after the adoptive transfer colitis model (*n*  = 5). (g) The percentages of CD4^+^ T cells in the spleen, pLN, mLN, and colon in (f) were determined (*n* = 5); results were analyzed by an unpaired *t*‐test. (h) DDX21 protein levels in mLN CD4^+^ T cells were measured in mice treated with 2.5% dextran sodium sulfate (DSS) for 4 days, followed by 4 days of normal drinking water. Data represent one of three independent experiments and are shown as the means ± SEM. ^**^ *p* < 0.01, ^***^ *p* < 0.001, ^****^ *p* < 0.0001.

To identify the role of DDX21 in regulating CD4^+^ T cells in IBD, we first employed adoptive transfer colitis, a well‐established T cell‐based IBD model [30]. When transferred to lymphodepleted mice, naïve CD4^+^ T cells typically undergo steady‐state expansion and differentiate into effector T cells, thereby triggering colitis [31]. This adoptive transfer model thus enables direct evaluation of the intrinsic pathogenic potential of a defined T cell subset, independent of confounding immune variables. Naïve CD4^+^ T cells were purified from WT and *Ddx21*‐cKO mice and transferred into *Rag1* ^−/−^ recipients. Recipients receiving WT naïve T cells began to lose weight in the fourth week post‐transplantation (Figure 3a). In contrast, mice receiving *Ddx21*–deficient naïve CD4^+^ T cells consistently gained weight throughout the experiment (Figure 3a) and had longer colons compared to those receiving WT naïve CD4^+^ T cells at 9 weeks post‐transplantation (Figure 3b,c). Compared to mice receiving WT naïve CD4^+^ T cells, mice receiving *Ddx21*–deficient naïve CD4^+^ T cells exhibited markedly reduced colitis symptoms (Figure 3d,e). Notably, while WT naïve CD4^+^ T cells established a stable population after 9 weeks of expansion, *Ddx21*–deficient CD4^+^ T cells were nearly eliminated from the recipients’ spleens, lymph nodes, and colons (Figure 3f,g). These data underscore that DDX21 is indispensable for the in vivo survival of activated CD4^+^ T cells.

To further evaluate the functional impact of DDX21 on T cells in vivo, we utilized the DSS‐induced colitis model, which disrupts the intestinal epithelial barrier to trigger inflammation and recapitulate key features of IBD [32]. This model is well‐suited for initial phenotypic screening and for evaluating the therapeutic potential of pharmacological interventions. To control for the potential influence of gut microbiota, DSS‐colitis experiments used co‐housed *Ddx21*‐cKO and WT mice, which indeed showed similar gut microbial profiles (Figure S5a,b).

We first examined DDX21 expression under inflammatory conditions. In DSS‐colitic mice, DDX21 was upregulated in CD4^+^ T cells from mesenteric lymph nodes (mLN) (Figure 3h), mirroring its elevated expression in CD4^+^ T cells from inflamed human IBD tissues (Figure 1b). By contrast, DDX21 levels were only modestly increased in macrophages and unchanged in dendritic cells within inflamed colons (Figure S6a,b). Notably, *Ddx21*‐cKO mice displayed a significant attenuation of DSS‐induced colitis compared to WT controls (Figure S6c–g). Although in vitro polarization assays revealed that *Ddx21*–deficient naïve CD4^+^ T cells exhibited a skewed differentiation profile, showing suppressed potential toward the Th17 lineage alongside enhanced differentiation into Th1, Th2, and iTreg subsets (Figure S7a–d), this altered differentiation pattern was not observed in vivo. In the DSS‐induced colitis model, we observed a pronounced reduction in the proliferation of colonic *Ddx2* *1*–deficient CD4^+^ T cells (Figure S6h,i), while their cytokine production showed no significant difference (Figure S6j). Furthermore, upon depletion of CD8^+^ T cells, *Ddx21*‐cKO mice still exhibited greater protection against colitis than WT mice (Figure S8a–e), underscoring a specific role for CD4^+^ T cells in this process. Collectively, these findings demonstrate that loss of DDX21 alleviates T cell‐driven colitis, primarily by restraining the expansion of CD4^+^ T cells.

To further clarify the role of DDX21 in T cell‐mediated autoimmune diseases, we utilized an experimental autoimmune encephalomyelitis (EAE) model, a classical T cell‐driven autoimmune disease, and a commonly used model for human inflammatory demyelinating diseases like multiple sclerosis [33, 34]. We found that the immunization of WT mice with the myelin oligodendrocyte glycoprotein (MOG) peptide MOG_35‐55_ induced significant EAE clinical scores (Figure S9a). In contrast, *Ddx21*‐cKO mice exhibited resistance to EAE induction, with reduced infiltration of CD4^+^ and CD8^+^ T cells into the central nervous system (CNS) (Figure S9b,c). These results indicate that the deletion of DDX21 in T cells confers protection against experimental neuroinflammation.

### 2.4 DDX21 Drives T Cell Proliferation via Cell Cycle Regulation

**FIGURE 4:**
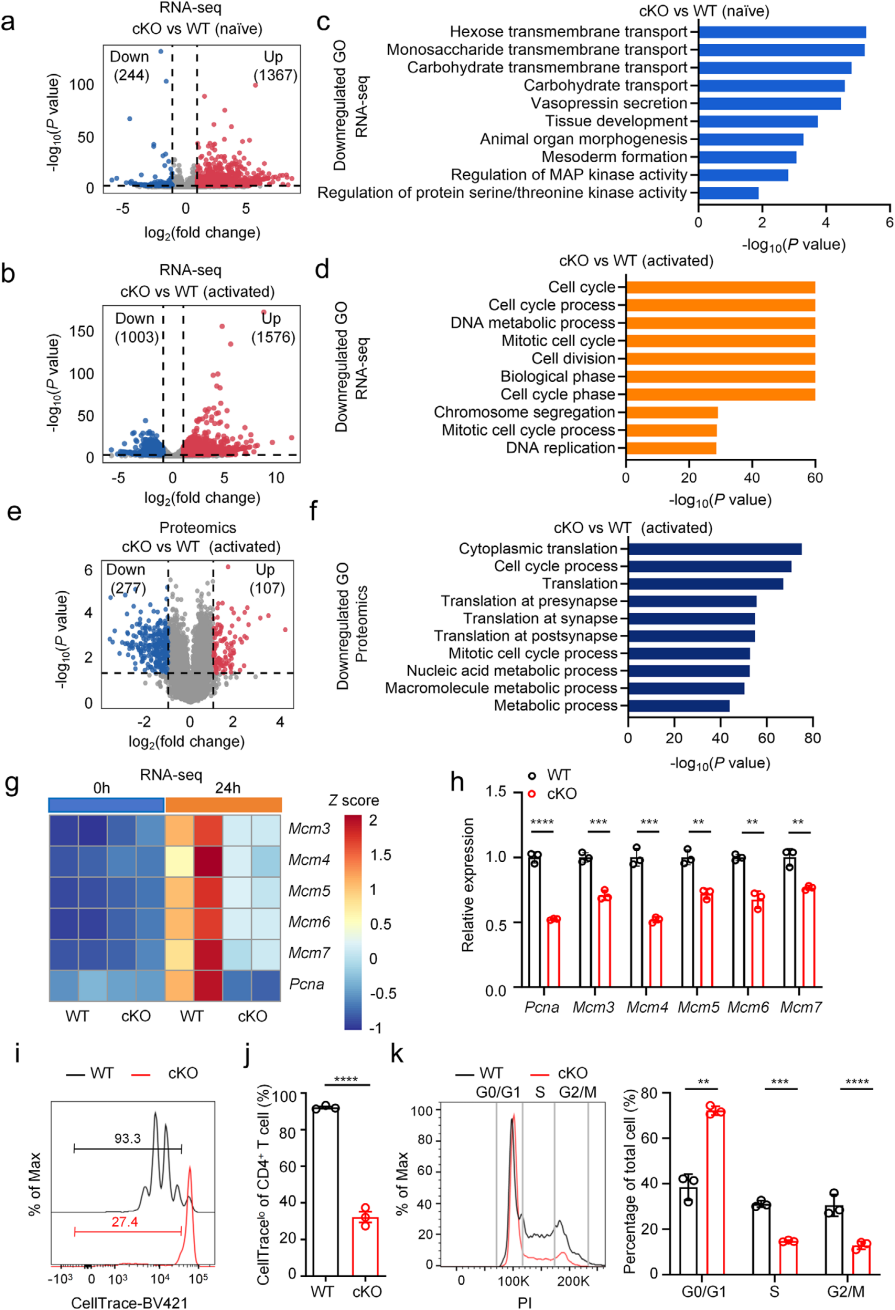
Multi‐omics profiling reveals impaired cell cycle in *Ddx21*–deficient CD4^+^ T cells. (a) Volcano plots of RNA sequencing data comparing *Ddx21*–deficient naïve CD4^+^ T cells with WT naïve CD4^+^ T cells. (b) Volcano plots of RNA sequencing data comparing *Ddx21*–deficient CD4^+^ T cells activated by anti‐CD3/CD28 antibodies for 24 hours with WT‐activated CD4^+^ T cells. (c) GO enrichment analysis of downregulated transcripts in *Ddx21*–deficient naïve CD4^+^ T cells compared to WT naïve CD4^+^ T cells. (d) GO enrichment analysis of downregulated transcripts in *Ddx21*–deficient CD4^+^ T cells activated by anti‐CD3/CD28 antibodies for 24 hours compared to WT‐activated CD4^+^ T cells. (e) Volcano plot depicting the differential protein expression in *Ddx21*–deficient CD4^+^ T cells activated by anti‐CD3/CD28 antibodies for 24 hours, compared to WT‐activated CD4^+^ T cells. (f) GO enrichment analysis showing the downregulated protein expression in *Ddx21*–deficient activated CD4^+^ T cells vs. WT‐activated CD4^+^ T cells. (g) Heatmap displaying the expression of transcripts related to the cell cycle and DNA replication pathways, with reduced expression in *Ddx21*–deficient activated CD4^+^ T cells compared to WT activated CD4^+^ T cells. (h) Real‐time qPCR measurements of levels of *Mcm3‐7* and *Pcna* mRNAs in *Ddx21*–deficient and WT naïve CD4^+^ T cells stimulated with anti‐CD3/CD28 antibodies for 24 hours; results were analyzed by an unpaired *t*‐test. (i,j) The proliferation of WT and *Ddx21*–deficient naïve CD4^+^ T cells was assessed in the presence of anti‐CD3/CD28 antibodies. Flow cytometry analysis (i) and ratios (j) of activated CD4^+^ T cells are shown as measured by CellTrace dilution after 3 days of activation; results were analyzed by an unpaired *t*‐test. (k) Naïve CD4^+^ T cells were isolated from *Ddx21*‐cKO and WT mice and activated with anti‐CD3/CD28 antibodies for 48 hours. Flow cytometry analysis of the cell cycle of activated CD4^+^ T cells is shown; results were analyzed by an unpaired *t*‐test. Data in (h–k) represent one of three independent experiments and are shown as the means ± SEM, *n* = 3 independent biological samples. ^**^ *p* < 0.01, ^***^ *p* < 0.001, ^****^ *p* < 0.0001.

To systematically explore the molecular mechanisms underlying this proliferative defect, we performed RNA‐sequencing analysis on naïve and in vitro activated CD4^+^ T cells (stimulated with anti‐CD3/CD28 antibodies for 24 hours) from *Ddx21*‐cKO mice and their WT littermates (Figure 4a,b). We found that downregulated GO pathways related to cell cycle and DNA replication were significantly enriched in activated *Ddx21*–deficient CD4^+^ T cells but not in naïve CD4^+^ T cells (Figure 4c,d). Additionally, proteomics results revealed that the cell cycle pathway was significantly downregulated in *Ddx21*‐cKO CD4^+^ T cells activated for 24 hours (Figure 4e,f). Further analysis of the RNA‐seq data from activated CD4^+^ T cells revealed that genes involved in DNA replication and cell cycle signaling pathways, such as *Mcm3‐7* and *Pcna* [35, 36], were significantly downregulated in *Ddx21*–deficient CD4^+^ T cells (Figure 4g). We further confirmed these findings by RT‐qPCR experiments (Figure 4h). These results suggest that *Ddx21*‐deficiency impairs CD4^+^ T cell proliferation by modulating the expression of transcripts related to DNA replication and cell cycle pathway genes during CD4^+^ T cell activation.

Given the substantial impairment in the proliferation of *Ddx2*1–deficient CD4^+^ T cells under steady‐state conditions, we hypothesized that the undetectable levels of *Ddx21*–deficient CD4^+^ T cells in the IBD model might also be attributed to this proliferative defect. To test this hypothesis, we conducted in vitro CD4^+^ T cell cultures by stimulating purified primary naïve CD4^+^ T cells with anti‐CD3/CD28 antibodies. Using the CellTrace labeling and dilution assay to assess the proliferative capacity of activated CD4^+^ T cells, we found that *Ddx21*–deficient CD4^+^ T cells exhibited significantly lower proliferative activity compared to their WT counterparts (Figure 4i,j). Since rapid T cell proliferation is closely linked to cell cycle progression upon activation [37, 38, 39], we further performed cell cycle analysis and measured DNA synthesis. Consistent with our hypothesis, the proportion of *Ddx21*–deficient CD4^+^ T cells in the S phase was reduced, while the proportion in the G0/G1 phase was markedly increased compared to WT control cells (Figure 4k). This indicates that cell cycle progression from the G0/G1 phase to the S phase is arrested in *Ddx21*–deficient CD4^+^ T cells. Collectively, these results suggest that DDX21 primarily facilitates the proliferation of CD4^+^ T cells after activation.

### 2.5 DDX21 Governs T Cell Ribosome Biogenesis and Global Translation Processes

**FIGURE 5:**
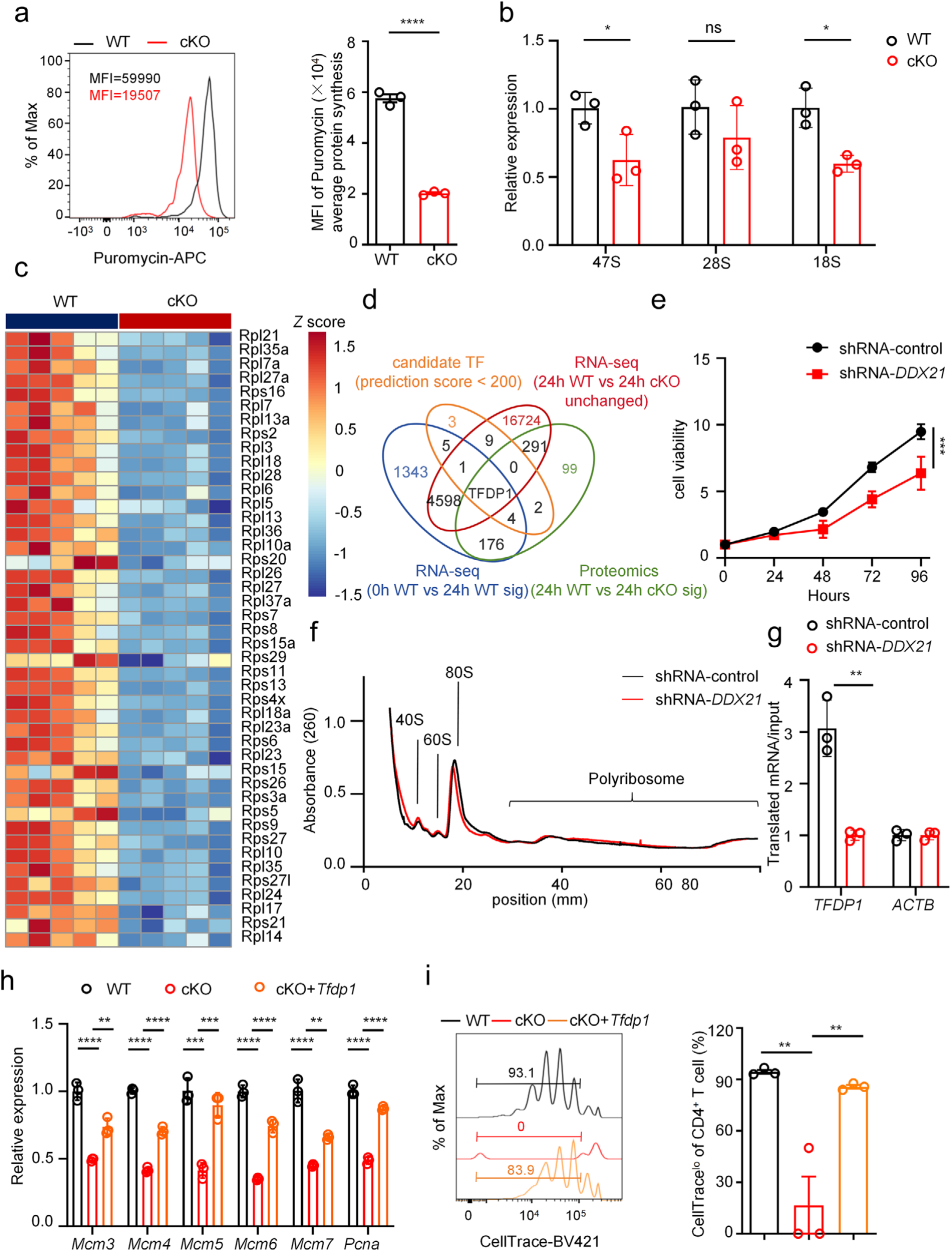
DDX21‐dependent translational control of TFDP1 drives cell cycle progression in CD4^+^ T cells. (a) Flow cytometry analysis of protein synthesis of activated CD4^+^ T cells, assessed by the mean fluorescence intensity (MFI) of O‐propargyl‐puromycin (*n* = 3); results were analyzed by unpaired *t*‐test. (b) Detection of rRNA expression in *Ddx21*–deficient CD4^+^ T cells vs. control CD4^+^ T cells, following 24 hours of activation with anti‐CD3/CD28 antibodies (*n* = 3); results were analyzed by unpaired *t*‐test. (c) Heatmap showing protein expression of ribosomal proteins in *Ddx21*–deficient CD4^+^ T cells vs. control CD4^+^ T cells. (d) Venn diagram showing the screening of transcription factors (criteria: changes in mRNA levels during T cell activation; no difference in mRNA levels between WT and *Ddx21*–deficient CD4^+^ T cells 24 hours after activation; changes in protein levels between WT and *Ddx21*–deficient CD4^+^ T cells 24 hours after activation; a ChEA3 prediction score < 200). (e) Cell viability of DDX21‐knockdown and WT Jurkat T cells was measured using the Cell Counting Kit‐8 (CCK‐8); results were analyzed by two‐way ANOVA (*n* = 3). (f) Representative trace of ribosome extract prepared from DDX21‐knockdown and WT Jurkat T cells in the presence of cycloheximide. The ribosomal extract was fractionated utilizing a 20%–60% sucrose gradient and analyzed with a pump syringe apparatus attached to a UV detector (*n* = 3). (g) Ribosome occupancy of *TFDP1* mRNAs was measured by real‐time PCR after sucrose gradient fractionation of polyribosomes; results were analyzed by unpaired *t*‐test (*n* = 3). (h) Real‐time PCR measurements of *Mcm3‐7* and *Pcna* mRNA levels in *Ddx21*‐cKO CD4^+^ T cells infected with TFDP1‐overexpressing lentivirus or empty vector control; results were analyzed by one‐way ANOVA (*n* = 3). (i) Flow cytometric analysis of CellTrace dilutions in TFDP1‐overexpressing lentivirus‐infected *Ddx21*‐cKO CD4^+^ T cells; results were analyzed by one‐way ANOVA (*n* = 3). Data represent one of three independent experiments and are shown as the means ± SEM, ^*^ *p* < 0.05, ^**^ *p* < 0.01, ^***^ *p* < 0.001, ^****^ *p* < 0.0001. NS, not significant.

Proteomics results also revealed that translation‐related pathways were significantly downregulated in *Ddx21*‐cKO CD4^+^ T cells activated for 24 hours (Figure 4e,f). To determine whether *Ddx21*‐deficiency impacts translation during CD4^+^ T cell activation, we analyzed the protein synthesis capacity of CD4^+^ T cells and found that *Ddx21*‐deficiency reduced translation levels in CD4^+^ T cells (Figure 5a), further confirming the role of DDX21 in translational regulation during CD4^+^ T cell activation.

Ribosome biogenesis is an important part of translation regulation, and eukaryotic ribosomes consist of different ribosomal RNAs (rRNAs) and ribosomal proteins, with contributions from all three RNA polymerases [40, 41]. Mutations in ribosomal proteins, reduced rDNA transcription, or altered rRNA processing can disrupt ribosome production [42]. DDX21 has been shown to regulate rDNA transcription and ribosome biogenesis [22], and it coordinates *Vegfc*‐driven developmental lymphangiogenesis by balancing endothelial cell ribosome biogenesis and p53 function [43]. To establish the effect of DDX21 on ribosome biogenesis, we measured the expression of rRNAs in CD4^+^ T cells and found that *Ddx21*‐deficiency suppressed 47S and 18S rRNAs compared with controls (Figure 5b). Heatmaps of the proteomics results showed that most of the ribosomal proteins were significantly reduced in *Ddx21*–deficient CD4^+^ T cells compared to WT cells (Figure 5c). We therefore selected RPS20, a representative ribosomal protein, as a marker for ribosome biogenesis and examined its expression. Compared with WT CD4^+^ T cells, both mRNA and protein levels of RPS20 were reduced in *Ddx21*–deficient CD4^+^ T cells (Figure 5c). We further evaluated the correlation between *DDX21* and *RPS20* expression using single‐cell RNA‐seq data from human IBD cohorts. Higher *DDX21* expression was positively correlated with increased *RPS20* expression (R  =  0.26, *P*  =  0.0091; Figure S10a), and this correlation was markedly stronger under inflammatory conditions (R  =  0.52, *P*  =  8.4 × 10^−6^; Figure S10a). Thus, these findings demonstrated that DDX21 is a positive regulator of ribosome biogenesis and global translation efficiency in CD4^+^ T cells.

### 2.6 DDX21 Enables TFDP1 Translation to Promote Cell Cycle Progression in CD4^+^ T Cells

Given that both RNA sequencing and proteomics analyses revealed disruption of the cell cycle pathway in *Ddx21*–deficient CD4^+^ T cells 24 hours post‐activation (Figure 4a–g), we employed the ChIP‐X Enrichment Analysis 3 (ChEA3) tool [44] (ChEA3, https://amp.pharm.mssm.edu/ChEA3) to identify potential transcription factors that may explain the defect of cell cycle and DNA replication pathways in *Ddx21*–deficient CD4^+^ T cells. The criteria for prediction included: dynamic mRNA expression changes during T cell activation; unaltered mRNA levels between WT and *Ddx21*–deficient CD4^+^ T cells at 24 hours post‐activation; differential protein expression in *Ddx21*–deficient vs. WT CD4^+^ T cells at 24 hours and a ChEA3 prediction score < 200. Surprisingly, after integrating these parameters, we only identified one transcription factor—TFDP1 (Figure 5d). We further predicted the interaction between DDX21 and *Tfdp1* mRNA through catRAPID [45] (Table S1, http://s.tartaglialab.com/page/catrapid_group). The results indicated a direct interaction between DDX21 and *Tfdp1* mRNA, which was subsequently validated using RNA immunoprecipitation (RIP)‐qPCR (Figure S10b). TFDP1 has been identified as a critical transcription factor for regulating the cell cycle and proliferation [46, 47], which aligns with the impaired proliferation phenotype observed in *Ddx21*–deficient CD4^+^ T cells.

Our previous multi‐omics analyses have shown that *Ddx21* deficiency in CD4^+^ T cells does not alter *Tfdp1* mRNA levels but significantly reduces TFDP1 protein expression. To further explore the potential impact of DDX21 on *Tfdp1* translation, we planned to conduct polyribosome profiling experiments. However, given the substantial reduction in CD4^+^ T cells in our *Ddx21‐*cKO mice and the high number of primary cells required for this experiment, we opted to use the Jurkat cell line as an alternative model. Jurkat cells, an acute T lymphoblastic leukemia cell line that expresses CD4, are commonly used for T cell functional validation experiments [48, 49, 50]. Our analysis using the single‐cell scRNA‐seq database TISCH2 [51] (Tumor Immune Single‐cell Hub 2, http://tisch.compbio.cn/home/) revealed that DDX21 is expressed at higher levels in proliferating T cells compared to conventional CD4^+^ T cells from ALL and CLL (Figure S10c). We then constructed DDX21‐knockdown Jurkat cells and found that reduced DDX21 expression did not affect *TFDP1* mRNA levels but decreased protein levels (Figure S10d,e). We further demonstrated that DDX21 downregulation led to decreased cell viability (Figure 5e) and increased early apoptosis (Figure S10f,g), consistent with our observations in *Ddx21*–deficient CD4^+^ T cells. Subsequently, we performed polyribosome‐quantitative RT‐qPCR experiments to measure the ribosome occupancy of TFDP1 mRNAs. Our results showed a significant reduction in ribosome accumulation on TFDP1 mRNA following DDX21 reduction, while control transcripts remained unaffected (Figure 5f,g). These findings suggest that DDX21 may influence T cell proliferation by regulating *TFDP1* mRNA translation.

To validate the contribution of TFDP1 to the observed phenotypes of *Ddx21*–deficient CD4^+^ T cells, we isolated naïve CD4^+^ T cells from WT and *Ddx21*‐cKO mice and overexpressed TFDP1 protein via lentivirus in CD4^+^ T cells stimulated with anti‐CD3/CD28 antibodies for 24 hours. Overexpression of TFDP1 increased the expression of cell cycle‐related genes in *Ddx21*–deficient CD4^+^ T cells (Figure 5h and Figure S10h,i). We subsequently evaluated the proliferative capacity of the infected CD4^+^ T cells and observed that TFDP1 overexpression nearly restored the proliferation of *Ddx21*–deficient CD4^+^ T cells to normal levels (Figure 5i). Consistent with our observations in mice, immunofluorescence analysis of IBD patient biopsies confirmed that DDX21, its downstream target TFDP1, and the ribosomal marker RPS20 were all elevated specifically in CD4^+^ T cells within inflamed intestinal tissues compared to non‐inflamed regions (Figure S10j–l). To further evaluate the in vivo role of TFDP1 in DDX21‐mediated CD4^+^ T cell function, we performed TFDP1 complementation experiments. Mice were treated with 2.5% DSS and transiently depleted of CD4^+^ T cells using an anti‐CD4 monoclonal antibody, followed by intravenous reintroduction of various modified CD4^+^ T cells 3 days afterward [52]. As expected, CD4^+^ T cell depletion alone resulted in colon elongation, indicating reduced colonic inflammation (Figure S11a–e). Notably, reintroduction of WT CD4^+^ T cells reversed this protective effect (Figure S11a–e). Compared with recipients of *Ddx21*–deficient CD4^+^ T cells, mice that received *Ddx21*–deficient CD4^+^ T cells overexpressing TFDP1 exhibited reduced body weight, colon shortening, elevated DAI scores, and more severe intestinal damage (Figure S11a–e). Moreover, the proportion of colonic CD4^+^ T cells was significantly increased (Figure S11f,g). Consistent with these findings, adoptive transfer of *Ddx21*–deficient CD4^+^ T cells overexpressing TFDP1 into *Rag1* ^−/−^ recipient mice also led to weight loss, colon shortening, increased DAI scores, and enhanced CD4^+^ T cell infiltration in the colon, compared with transfer of *Ddx21*–deficient CD4^+^ T cells (Figure S11h–n). Collectively, these results demonstrate that TFDP1 complementation reverses the protective phenotype conferred by DDX21 deficiency, supporting TFDP1 as a critical downstream effector in DDX21‐mediated colitis pathogenesis.

### 2.7 Pharmacological Inhibition of Ribosome Biogenesis Alleviates T Cell‐Driven Colitis

**FIGURE 6:**
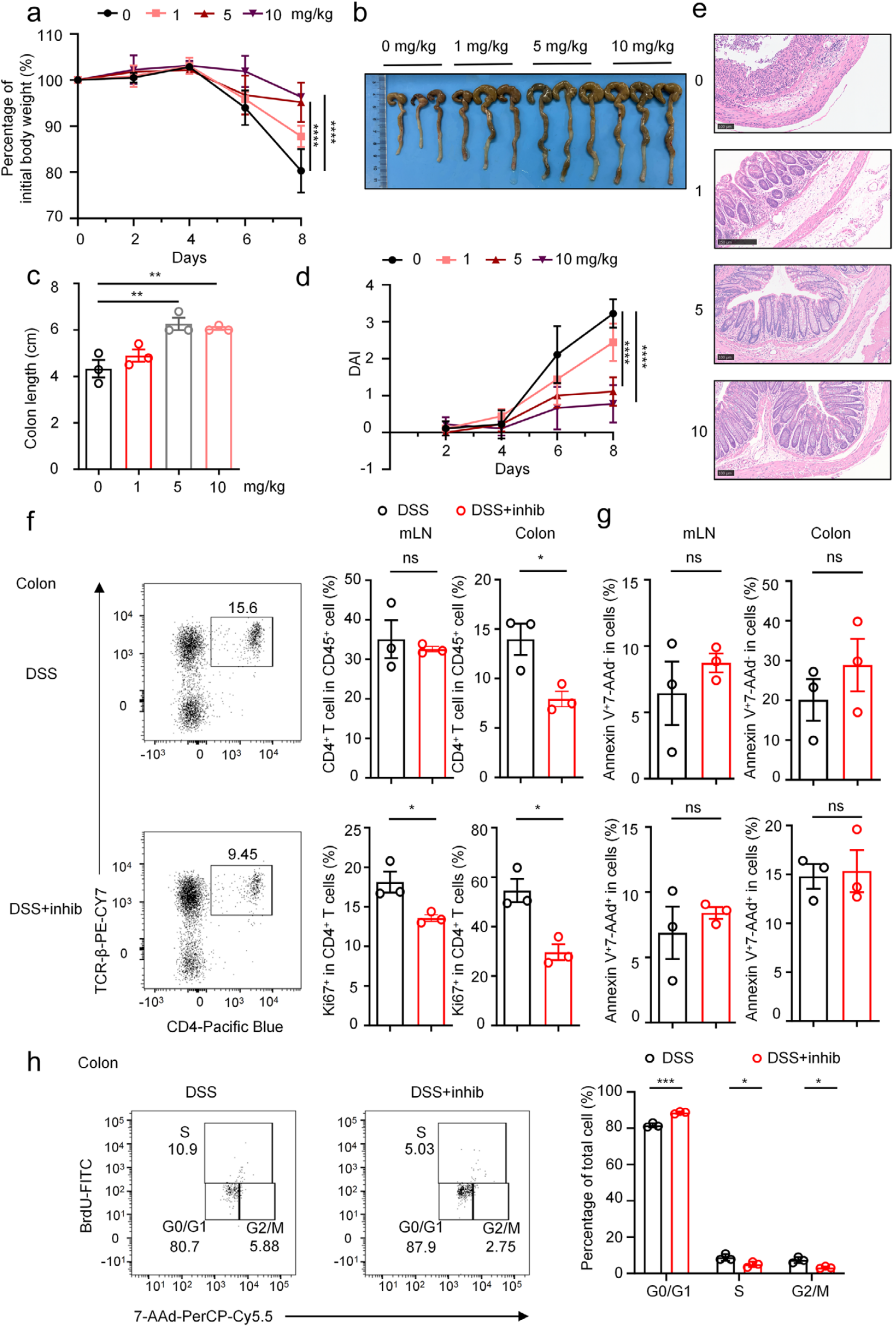
Pharmacological inhibition of ribosome biogenesis alleviates T cell‐driven colitis. (a) Mice received 2.5% DSS for 4 days followed by 4 days of normal water and were administered different doses of KU55933 twice weekly; body weight was monitored every 2 days, and results were analyzed by two‐way ANOVA (*n* = 3). (b,c) Representative colon images (b) and length measurements (c) in (a) at day 8 post‐treatment (*n* = 3); results were analyzed by one‐way ANOVA. (d) The DAI of mice was analyzed by two‐way ANOVA (*n* = 3). (e) Representative images of H&E staining of the colons on day 8 (*n*  = 3); scale bar = 100 µm. (f) Flow cytometric analysis of colonic CD4^+^ T cell composition in mice treated with 5 mg/kg KU55933 at day 8. The percentages of CD4^+^ T cells and Ki67^+^ CD4^+^ T cells from the mLN and colon of mice were determined (*n* = 3). (g) Ratios of Annexin V^+^ 7‐AAD^−^ and Annexin V^+^ 7‐AAD^+^ cells are shown (*n* = 3); results were analyzed by an unpaired *t*‐test. (h) Flow cytometric analysis of CD4^+^ T cells in the colon of mice treated or untreated with 5 mg/kg KU55933 was performed at day 8 (*n* = 3); results were analyzed by an unpaired *t*‐test. Data represent one of three independent experiments and are shown as the means ± SEM. ^*^ *p* < 0.05, ^**^ *p* < 0.01, ^***^ *p* < 0.001, ^****^ *p* < 0.0001. NS, not significant.

Given that DDX21 sustains T cell proliferation through ribosome biogenesis (Figure 5b,c), we hypothesized that pharmacological inhibition of this pathway might recapitulate the protective effects of *Ddx21* deletion. We employed KU55933, a compound reported to suppress ribosome biogenesis‐related signaling via ATM kinase inhibition [53, 54, 55]. Compared with WT CD4^+^ T cells, *Ddx21*–deficient CD4^+^ T cells had lower ATM and phospho‐ATM levels. KU55933 treatment during CD4^+^ T cell activation also reduced ATM and phosphorylated ATM levels (Figure S12a,b). Moreover, KU55933 inhibited the proliferation activity of CD4^+^ T cells in vitro and had minimal effects on the viability of macrophages and epithelial cells (Figure S12c). KU55933 inhibited protein synthesis and cell cycle progression in CD4^+^ T cells, mirroring the phenotype observed in *Ddx21*–deficient T cells (Figure S12d,e). We first evaluated the potential toxicity of KU55933 in vivo. Different doses of KU55933 were administered to WT mice via intraperitoneal injection twice weekly, and all mice were euthanized after 7 days. Compared to the control group, there were no significant changes in the body weight or colon length of the drug‐treated group (Figure S13a–c). Moreover, the compound showed no notable effects on organ weight or histology of the liver, spleen, or kidney (Figure S13d, e). Plasma biochemical markers—aspartate aminotransferase (AST), alanine aminotransferase (ALT), blood urea nitrogen (BUN), and creatinine (Cr)—were largely unchanged (Figure S13f). Thus, short‐term exposure to KU55933 showed no marked toxicity. Strikingly, KU55933 administration ameliorated DSS‐induced colitis in a dose‐dependent manner (Figure 6a–e). Flow cytometry further showed that KU55933 reduced both the infiltration of CD4^+^ T cells into the colon lamina propria and the frequency of proliferating (Ki67^+^) CD4^+^ T cells (Figure 6f). Importantly, this suppression of pathogenic CD4^+^ T‐cell expansion resulted from cell‐cycle arrest, not from increased apoptosis (Figure 6g,h), thereby phenocopying the proliferative defect observed in *Ddx21*–deficient T cells. Collectively, these data demonstrate that pharmacologically targeting DDX21‐associated ribosome biogenesis effectively mitigates T cell‐mediated pathology in experimental IBD.

## 3 Discussion

T cell activation and proliferation necessitate bypassing multiple molecular checkpoints [56, 57], with RBPs playing critical regulatory roles [58, 59]. Our prior research identified TRMT61A‐mediated m^1^A58 tRNA modification as a translational checkpoint controlling CD4^+^ T cell proliferation [29]. However, this study reveals that the RNA helicase DDX21 governs CD4^+^ T cell expansion through a distinct layer of translational control.

DEAD‐box RNA helicases, including DDX21, are fundamental regulators of RNA metabolism, encompassing processes such as transcription, splicing, translation, and degradation [60]. While several DEAD‐box helicases, like DDX41 and DDX3X, have established roles in antiviral immune responses by sensing nucleic acids and modulating signaling pathways [61, 62], and others like DDX1 and DDX5 are implicated in tumor immune evasion or cancer cell proliferation [63, 64], the specific functions of DDX21 in adaptive immunity, particularly in T cell biology, have been less defined.

Using integrated genetic, biochemical, and multi‐omics approaches (RNA‐seq and proteomics), we demonstrate that DDX21 is essential for the proliferation and homeostasis of CD4^+^ T cells, particularly in inflammatory contexts, as evidenced by the correlation between *DDX21* expression and cell‐cycle pathways in CD4^+^ T cells from IBD‐inflamed tissues. Mechanistically, DDX21 sustains ribosome biogenesis and global translation upon T cell activation. *Ddx21* deficiency disrupts this process, reducing ribosomal protein/rRNA expression and impairing the translational machinery necessary for expansion. Consequently, *Ddx21* ^−/−^ CD4^+^ T cells exhibit diminished cell‐cycle progression and attenuated pathogenicity in adoptive transfer colitis. We further identified TFDP1—a transcription factor vital for cell‐cycle progression—as a key translational target of DDX21. Reduced TFDP1 expression in *Ddx21* ^−/−^ T cells contributes to the proliferation defect, a conclusion supported by the near‐complete rescue of proliferation upon TFDP1 overexpression (Figure S14).

The correlation between DDX21 expression and IBD severity underscores its therapeutic potential for T cell‐mediated diseases, where modulating DDX21 activity could control pathological T cell expansion in autoimmune disorders. However, key questions remain: while we mapped downstream consequences of DDX21 deficiency, upstream regulators of its expression in T cells are unknown, necessitating future studies on control mechanisms during T cell activation and pathogenic processes. Furthermore, while our work focuses on CD4^+^ T cells, DDX21 also regulates CD8^+^ T cell homeostasis via non‐proliferative pathways. Although we have identified DDX21's specific role in CD4^+^ T cell‐mediated colitis, further investigation into its regulatory functions in CD8^+^ T cells and other immune compartments merits dedicated future investigation. The altered differentiation pattern of *Ddx21*–deficient CD4^+^ T cells also suggests that DDX21 may influence T cell fate decisions, meriting further study.

Critically, elevated DDX21 in colitis‐affected human/murine CD4^+^ T cells, concomitant with KU55933‐mediated alleviation of acute DSS‐induced colitis, validates its therapeutic relevance; KU55933 phenocopies the protective effects of *Ddx21* deletion by suppressing pathogenic CD4^+^ T cell expansion without apoptosis, establishing ribosome biogenesis inhibition as a viable strategy. These findings collectively establish a proof‐of‐concept for targeting ribosome biogenesis in T cell immune pathologies. However, our work was limited to an acute colitis model. Whether it affects long‐term normal immune responses requires further observation. Subsequent research is needed to verify whether its protective effects can be extended to chronic models and subsequent complications (such as tumorigenesis and tissue fibrosis). Moreover, the translational potential of this strategy in human patients requires validation. The multifaceted effects and protective role of KU55933 stem from its comprehensive inhibition of ATM—a mechanism not solely attributable to mimicking *Ddx21* deficiency. Its protective effects may involve multiple aspects, potentially extending beyond T cell specificity. There is an urgent need to develop more specific DDX21‐targeted therapeutic agents.

In conclusion, our findings establish DDX21 as a critical regulator of CD4^+^ T cell proliferation through ribosome biogenesis and selective control of TFDP1 translation. This expands the paradigm of post‐transcriptional checkpoints in T cell immunity and opens avenues for targeting RNA helicases in immune‐mediated diseases. Future work should address the unresolved mechanistic and translational aspects to fully exploit DDX21's therapeutic potential.

## 4 Methods

### 4.1 Mice

*Ddx21* ^flox/flox^ mice were genetically engineered using the CRISPR/Cas9 system, which involved the insertion of two loxP sites flanking the exons 3–5 by Cyagen Biosciences Inc. *Cd4* ^Cre^ mice were procured from the Jackson Laboratory. *Cd4* ^Cre^ mice were crossed with *Ddx21* ^flox/flox^ mice to produce *Ddx21* ^flox/flox^ *Cd4* ^Cre^ offspring. The sex‐matched *Ddx21* ^flox/flox^ offspring lacking *Ddx21* expression in CD4^+^ T cells were used as cKO mice, with *Ddx21* ^flox/flox^ mice serving as WT controls. All mice were bred and maintained under specific pathogen‐free conditions. Both male and female mice were used at 8 to 12 weeks of age to carry out experiments. Animals were randomly divided into experimental groups, and each cage housed mice from different experimental groups. The Institutional Animal Care and Use Committee (IACUC) of Shanghai Jiao Tong University School of Medicine approved all animal procedures (No. JUMC2023‐086‐A).

### 4.2 Cell Isolation

The spleen, thymus, and lymph node were extracted by pressing through a 200‐mesh strainer. Erythrocytes were subsequently lysed with erythrocyte lysis buffer (Thermo Fisher Scientific) to prepare splenic single‐cell leukocyte suspensions. The CNS (including the brain and spinal cord) was removed and pressed with a 200‐gauge mesh. CNS mononuclear cells were then collected following density gradient centrifugation with 40% Percoll (Cytiva, no. 17089109).

Colon tissue was minced and sequentially digested to isolate different cell fractions. First, epithelial cells were released by digestion in a solution containing 5 mm EDTA and 1 mm DTT, then collected by filtration through a 200‐mesh strainer for culture. Subsequently, the remaining tissue was further digested with a solution of 1 mg/mL collagenase II and 0.5 mg/mL Dispase. The resulting digest was filtered through a 200‐mesh strainer, and colonic mononuclear cells were finally isolated by density gradient centrifugation using 40% Percoll.

### 4.3 Antibody Staining and Flow Cytometry

Monoclonal antibodies targeting CD45 (30‐F11), CD3 (145‐2C11), CD4 (RM4‐5), CD8 (53‐6.7), TCR‐β (H57‐597), CD44 (IM7), CD62L (MEL‐14), IFN‐γ (XMG1.2), Ki67 (clone 16A8), MHC‐II (M5/114.15.2), CD11b (M1/70), F4/80 (BM8), CD11c (N418) and PE anti‐ATM Phospho (Ser1981) were purchased from BioLegend (San Diego, CA, USA). DDX21 and ATM antibodies were purchased from Novus Biologicals. The isolated cells were incubated with rat serum to block Fc receptors, followed by labeling with fluorescently conjugated antibodies. For intracellular cytokine detection, cells were stimulated with phorbol 12‐myristate 13‐acetate (50 ng/mL; Sigma‐Aldrich, St. Louis, MO, USA) and ionomycin (1 µg/mL; Sigma–Aldrich) in the presence of GolgiPlug (1:1000; BD Biosciences, San Jose, CA, USA) for 4 hours. After surface staining, cells were fixed and permeabilized with a Fixation/Permeabilization Solution Kit (BD Biosciences) and then stained with antibodies against intracellular molecules. For intracellular transcription factor staining, cells were fixed and permeabilized with a Foxp3/Transcription Factor Buffer Set (eBioscience). BrdU/7‐AAD cell cycle staining was performed according to the instructions for the FITC BrdU Flow Kit (BD Biosciences). All data were collected with BD Fortessa X20 and analyzed with FlowJo software.

### 4.4 Adoptive Transfer Colitis

Naïve CD4^+^ T cells were purified from the spleen using the EasySep Mouse Naïve CD4^+^ T Cell Isolation Kit. A total of 5 × 10^5^ naïve CD4^+^ T cells from *Ddx21*‐cKO mice and WT littermate control mice were transferred into *Rag*1^−/−^ mice. The body weight of the recipient mice was monitored on a weekly basis.

### 4.5 Induction and Assessment of Experimental Autoimmune Encephalomyelitis (EAE)

*Ddx21*‐cKO mice and their WT littermate controls were subcutaneously injected with 200 µg of mouse MOG_35–55_ peptide (Prespec), which was emulsified in complete Freund's adjuvant containing heat‐killed Mycobacterium tuberculosis H37RA (BD Difco). Pertussis toxin (200 ng; List Biological Laboratories) was administered intravenously on the day of immunization and again 2 days post‐immunization. Subsequently, the mice were monitored daily and assessed using the following clinical score criteria: 0, no clinical signs; (1), limp tail; (2), paraparesis (weakness, incomplete paralysis of one or two hind limbs); (3), paraplegia (complete paralysis of two hind limbs); (4), paraplegia with forelimb weakness or paralysis; and (5), moribund or death, as previously described [65].

### 4.6 Dextran Sodium Sulfate (DSS)‐Induced Colitis

DSS‐induced colitis in mice is the most commonly used model for analyzing the pathogenesis of IBD. Studies indicate that mice should be administered DSS at concentrations of 2.5%–10% for 3–7 consecutive days to establish this model [66, 67, 68, 69]. Colitis was induced by adding 2.5% DSS (MW: 36 000–50 000; Yeasen) in the drinking water for 4 days, after which the animals were given normal drinking water. Mice aged 8–12 weeks were used. KU55933 (cat. no. sc‐202963, Santa Cruz Biotechnology) was administered via injection twice weekly to C57BL/6 mice, and all mice were euthanized 7 days later. For each mouse, the scores for weight loss, stool consistency, and fecal blood were summed, and the final DAI value was calculated as the mean of these three scores.

### 4.7 Histological Analysis

After the colitis model was established, mice were sacrificed, and their colons were fixed with 4% paraformaldehyde and embedded in paraffin. Serial paraffin sections were stained with hematoxylin and eosin to assess tissue inflammation.

### 4.8 T Cell Isolation and Stimulation

Naïve CD4^+^ T cells were purified from the spleens using the EasySep Mouse Naïve CD4^+^ T Cell Isolation Kit (STEMCELL Technologies). The purified T cells were stimulated with plate‐bound anti‐CD3 (1 µg/mL) and anti‐CD28 (2 µg/mL) antibodies in replicate wells of 96‐well plates. Naïve CD4^+^ T cells labeled with CellTrace (Thermo Fisher Scientific, cat. no. C34557) were stimulated with anti‐CD3 (1 µg/mL) and anti‐CD28 (2 µg/mL) antibodies for 72 hours. Cell proliferation was assessed and analyzed through CellTrace dilution. The apoptotic state of the cells was evaluated using the APC/PE Annexin V Apoptosis Detection Kit with 7‐AAD (BioLegend). Cells that were annexin V‐positive or annexin V/7‐AAD double‐positive were classified as apoptotic, whereas double‐negative cells were considered viable.

### 4.9 T Cell Ex Vivo Differentiation

Naïve CD4^+^ T cells were purified from the spleens by using the EasySep Mouse Naïve CD4^+^ T Cell Isolation Kit (STEMCELL Technologies) and cultured with anti‐CD3 mAb (1 µg/mL) and anti‐CD28 (2 µg/mL) antibodies in the presence of defined mouse recombinant cytokines and blocking antibodies. In brief, Th1 was induced with IL‐2 (2 ng/mL), IL‐12 (10 ng/mL) and anti‐IL‐4 mAb (10 µg/mL; 11B11); Th2 was induced with IL‐2 (5 ng/mL), IL‐4 (10 ng/mL) and anti‐IFN‐γ mAb (1 µg/mL; XMG1.2); Th17 was induced with IL‐6 (40 ng/mL), TGF‐β (2 ng/mL), anti‐IL‐4 mAb (10 µg/mL; 11B11) and anti‐IFN‐γ mAb (10 µg/mL; XMG1.2); Treg was induced with IL‐2 (2 ng/mL), TGF‐β (1 ng/mL). All cytokines were purchased from R&D Systems.

### 4.10 RNA Library Preparation, Sequencing, and Differentially Expressed Genes Analysis

CD4^+^ T cells were sorted from *Ddx21*‐cKO and WT mice, and total RNA was extracted using the TRIzol reagent (Invitrogen) following the manufacturer's instructions. RNA purity and quantification were measured using the NanoDrop 2000 spectrophotometer (Thermo Fisher Scientific, USA). Libraries were prepared with 1 µg of total RNA and sequenced on the Illumina NovaSeq 6000 platform by Shanghai Neo‐Biotechnology Co., Ltd (Shanghai, China), producing 150 bp paired‐end reads. For each sample, STAR software was used to align the preprocessed sequences with the reference genome sequences of the sequenced species. StringTie software was used to count the raw sequence counts of the known genes and calculate the FPKM value for each gene. HTSeq‐count provided gene read counts. Principal component analysis (PCA) was performed with the R package to assess biological reproducibility between samples. DESeq2 was used to normalize the raw counts and identify differentially expressed genes (DEGs). Volcano plots and bar plots were generated using the R package. Kyoto Encyclopedia of Genes and Genomes pathway enrichment analysis of DEGs was performed using the R package. GO enrichment analysis of differentially expressed genes was performed with the R package.

### 4.11 Reverse Transcription qPCR

Total RNA was isolated from CD4^+^ T cells with TRIzol reagent (Invitrogen) in accordance with the manufacturer's instructions. The RNA was subsequently reverse transcribed using HiScript III RT SuperMix for qPCR (Vazyme, Cat: R323‐01). All RT‐qPCRs were conducted on a Bio‐Rad CFX96 real‐time system using ChamQ Universal SYBR qPCR Master Mix (Vazyme, Cat: Q711‐03). β‐actin was used as an internal control to normalize the data across different samples.

Primer sequences used for RT‐qPCR are as follows:

*Actb* (forward, 5′‐AGTGTGACGTTGACATCCGT‐3′; reverse, 5′‐GCAGCTCAGTAACAGTCCGC‐3′), ACTB (forward, 5′‐TGCGTCTGGACCTGGCTGGC‐3′; reverse, 5′‐GCCTCAGGGCAGCGGAACCG‐3′), *Ddx21* (forward, 5′‐GAGGCCGTTTCCTCCAAAG‐3′; reverse, 5′‐GTAGAAGCAGTGTCGTCTTGAG‐3′), *Tfdp1* (forward, 5′‐TTGAAGCCAACGGAGAACTAAAG‐3′; reverse, 5′‐TGGACTGTCCGAAGGTTTTTG‐3′), *Pcna* (forward, 5′‐TTGCACGTATATGCCGAGACC‐3′; reverse, 5′‐GGTGAACAGGCTCATTCATCTCT‐3′), *Mcm6* (forward, 5′‐ACCAACCCAAGGTTTGGAGG‐3′; reverse, 5′‐TAATGCTCTCAGCGGTCTGTT‐3′), *Mcm3* (forward, 5′‐AGCGCAGAGAGACTACTTGGA‐3′; reverse, 5′‐CAGCCGATACTGGTTGTCACT‐3′), *Mcm5* (forward, 5′‐GGGCATTTTCTACAGCGACAG‐3′; reverse, 5′‐GAACTCCTTGAATCGCCTCTG‐3′), *Mcm7* (forward, 5′‐AGTATGGGACCCAGTTGGTTC‐3′; reverse, 5′‐GCATTCTCGCAAATTGAGTCG‐3′), *Mcm4* (forward, 5′‐TCTTTGACCGTTATCCTGACTCC‐3′; reverse, 5′‐TGCCTCGATCTATCTCCACCC‐3′), *TFDP1* (forward, 5′‐CTTCCGACTCCTCACCTTGG‐3′; reverse, 5′‐AAATGCCGTAGGCCCTTGC‐3′), *DDX21* (forward, 5′‐ GAGGAGCCATCTCAAAATGACA‐3′; reverse, 5′‐ GGGTTACAGTCCGGTTCAGG‐3′),

47S (forward, 5′‐TTTTTGGGGAGGTGGAGAGTC‐3′; reverse, 5′‐CTGATACGGGCAGACACAGAAC‐3′), 28S (forward, 5′‐TGGTGTATGTGCTTGGCTGAGG‐3′; reverse, 5′‐CTGGGCGGGATTCTGACTTAGAG‐3′), 18S (forward, 5′‐CGGACACGGACAGGATTGACAG‐3′; reverse, 5′‐TGCCAGAGTCTCGTTCGTTATCG‐3′).

### 4.12 DirectDIA Quantitative Proteomics

The sample preparation for directDIA quantitative proteomics involved several key steps, including protein extraction, denaturation, reduction, alkylation, tryptic digestion, and peptide cleanup. A commercially available iST Sample Preparation kit (PreOmics, Germany) was used according to the protocols provided. The Vanquish Neo UHPLC system (Thermo Fisher Scientific, MA, USA)was connected to the timsTOF HT, an ion‐mobility spectrometry quadrupole time of flight mass spectrometer (Bruker Daltonik, Bremen, Germany). Samples were reconstituted in 0.1% formic acid (FA), and 200 ng of peptide was separated by the AUR3‐15075C18 column. Data‐independent acquisition (DIA) data were acquired in the diaPASEF mode by ChiBiotech Co., Ltd. The raw DIA Data were processed and analyzed by Spectronaut 19 (Biognosys AG, Switzerland) with default settings. PCA was carried out separately on each data set using the R function prcomp () from the stats package with default parameters. The volcano plot was drawn by using the ggplot2 package. The GO annotation was obtained from the Uniprot database, and Fisher's exact test was used to perform GO enrichment analysis.

### 4.13 RNA Immunoprecipitation (RIP) ‐qPCR

Total RNA extracted from spleen cells was purified. The purified mRNA was incubated with anti‐DDX21 antibody (Proteintech) or rabbit IgG in immunoprecipitation (IP) wash buffer (10 mm Tris‐HCL, 0.1% NP‐40, and 150 mm NaCl supplemented with ribonuclease inhibitor (pH 7.4) for 2 hours at 4°C. Protein A beads (Thermo Fisher Scientific, no. 21348) were added and incubated at 4°C for 2 hours. After incubation, the immunoprecipitated beads‐DDX21 antibody‐mRNA complex was extensively washed with IP wash buffer. TRIzol reagent (Invitrogen) was added to elute RNA. All qPCRs were run on the Bio‐Rad CFX96 real‐time system.

### 4.14 16S rDNA Amplicon Sequencing

16S rDNA Amplicon Sequencing employed universal primers designed for conserved regions to perform PCR amplification, followed by sequencing analysis and species identification of the hypervariable regions. Libraries were constructed based on amplified regions and underwent paired‐end sequencing by Novogene. Sequencing libraries were generated, and indexes were added. The library was checked with Qubit and real‐time PCR for quantification and a bioanalyzer for size distribution detection. Quantified libraries were pooled and sequenced on NovaSeq6000 or DNBSEQ‐G99 platforms, according to effective library concentration and data amount required. After reads assembly and filtering, OTUs (Operational Taxonomic Units) were clustered, or ASVs (Amplicon Sequence Variants) were denoised. The resulting valid data undergo species annotation and abundance analysis. Paired‐end reads were merged using FLASH (V1.2. 1 1, http://ccb.jhu.edu/software/FLASH/), a very fast and accurate analysis tool, which was designed to merge paired‐end reads when at least some of the reads overlap the read generated from the opposite end of the same DNA fragment, and the splicing sequences were called raw tags. Quality filtering on the raw tags was performed using the fastp (Version 0.23.1) software to obtain high‐quality Clean Tags.

### 4.15 Immunofluorescence Microscopy

Paraffin sections were obtained from the First Affiliated Hospital of Chongqing Medical University. Approval was granted by the Ethics Committee of the First Affiliated Hospital of Chongqing Medical University (2024‐543‐01), and informed consent was obtained from the patient. Paraffin sections were dewaxed with xylene (two times, 10 minutes each), rehydrated with a gradient ethanol series (100%, 95%, 85%, 75%; 5 minutes each) for rehydration, and finally rinsed with PBS. Slides were immersed in 10 mm sodium citrate buffer (pH 6.0) and heated at 95°C for 30 minutes for antigen retrieval. Sections were cooled to room temperature for 30 minutes. Three washes with PBS were performed, each lasting 5 minutes. Sections were blocked at room temperature with QuickBlock Blocking Buffer for Immunostaining (Beyotime, no. P0260) for 15 minutes. Sections were incubated overnight at 4°C with diluted primary antibody: DDX21 (Proteintech, no. 10528‐1‐AP, 1:100), TFDP1 (Invitrogen, no. PA5‐86135, 1:100), CD4 (Invitrogen, no. 740028T20UG, 1:800) and RPS20 (Invitrogen, no. MA5‐35806, 1:100). After three washes with PBS (30 minutes each), sections were incubated at room temperature in the dark with diluted fluorescently labeled secondary antibody for 1 hour. After three PBS washes, DAPI Fluoromount‐G (catalog no. 36308ES11, Yeasen) was used as the mounting medium. Slides were stored at 4°C in the dark until imaging. Image acquisition was performed using PanoBrain.

### 4.16 Biochemical Assays

Plasma samples from mice were collected in EDTA‐anticoagulated tubes. The activities of aspartate aminotransferase (AST), alanine aminotransferase (ALT), and the concentrations of blood urea nitrogen (BUN) and creatinine (Cr) were quantified using available assay kits (Nanjing Jiancheng Biological Engineering Institute, Cr: C011‐2‐1, BUN: C013‐2‐1, AST: C010‐2‐1, ALT: C009‐2‐1) in accordance with the manufacturer's protocols.

### 4.17 Macrophage Culture

Bone marrow‐derived macrophages (BMDMs) were prepared from bone marrow cells collected from the femur of the hind limb. Cells were cultured for 5 days in 10 cm untreated culture dishes using RPMI 1640 medium supplemented with 10% fetal bovine serum, 50 U/mL penicillin, 50 mg/mL streptomycin, and 100 ng/mL M‐CSF (Catalog No. 576408, Biolegend). Differentiated BMDMs were harvested and seeded into untreated sterile 96‐well tissue culture plates.

### 4.18 Western Blot

CD4^+^ T cells from lymphoid nodes were purified by the EasySep Mouse CD4^+^ T Cell Isolation Kit. Total protein was extracted from these CD4^+^ T cells utilizing radioimmunoprecipitation assay lysis buffer (Beyotime, no. P0013E) supplemented with ProteinSafe Protease Inhibitor Cocktail (Epizyme, no. GRF101) and Phosphatase Inhibitor Cocktail (Epizyme, no. GRF102). Antibodies against DDX21 (Proteintech, no. 10528‐1‐AP), and TFDP1 (Invitrogen, no. PA5‐86135) were diluted in 5% nonfat milk buffer at a concentration of 1:1000 and incubated overnight at 4°C. After washing the membrane three times with 0.1% TBST buffer, the horseradish peroxidase‐conjugated secondary antibody was added to the membrane and incubated at room temperature for 1 hour. The final signal was detected by Meilunbio fg super sensitive ECL luminescence reagent, and β‐Actin (Cell Signaling Technology, cat. no.3700S) was used as the internal control.

### 4.19 CCK‐8 Assay

For endpoint detection, CCK‐8 reagent was added to each well. The plate was returned to the incubator for 1–4 hours, and the absorbance at 450 nm was measured using a microplate reader.

### 4.20 Protein Synthesis Assay

To visualize protein synthesis at the single‐cell level, the O‐propargyl‐puromycin (OPP) labeling method was used, followed by Click‐iT chemistry detection of nascent peptides (Click‐iT Plus OPP Alexa Fluor 594 Protein Synthesis Assay Kit, Molecular Probes, C10457). Naïve CD4^+^ T cells were stimulated with plate‐bound anti‐CD3 (1 µg /mL) and anti‐CD28 (2 µg/ mL) for 24 hours. The cells were incubated in the Click‐iT OPP working solution for 30 minutes, followed by fixed permeabilization at room temperature. The cells were then incubated in the Click‐iT Reaction Cocktail for 30 minutes, and protein synthesis was finally detected via flow cytometry.

### 4.21 Polyribosome RT‐qPCR

We performed the ribosome profiling by strictly following the manual for Eukaryotic Polyribosome Profile Analysis. A total of 100 million DDX21‐knockdown (shRNA‐DDX21‐S: ATATCTTGTGGAAAGGACGAAAGCGGAGTTTCAGTAAAGCATTTTCAAGAGAAATGCTTTACTGAAACTCCGCTCGACCTCGAGACAAATGGCAGTATTC, shRNA‐DDX21‐AS: GAATACTGCCATTTGTCTCGAGGTCGAGCGGAGTTTCAGTAAAGCATTTCTCTTGAAAATGCTTTACTGAAACTCCGCTTTCGTCCTTTCCACAAGATAT, pLOK1 vector) and WT Jurkat T cells were washed, and subsequently exposed to 100 µg/mL cycloheximide for 1 minute. Following cell lysis, one‐third of the lysate was allocated for input RNA analysis, while the remaining portion was utilized for polyribosome preparation via centrifugation through 20%–60% sucrose gradients. The gradients were centrifuged at 274 000 g for 2 hours at 4°C, after which polyribosome fractions were collected. Both input RNAs and polyribosome RNAs were isolated, and the efficiency of gene translation was assessed using RT‐qPCR.

### 4.22 TFDP1 Overexpression in CD4^+^ T Cells

Naïve CD4^+^ T cells were isolated from WT or *Ddx21*‐cKO mice using the EasySep Mouse Naïve CD4^+^ T Cell Isolation Kit (STEMCELL Technologies). Cells were activated with anti‐CD3 /CD28 antibodies for 24 hours. The coding DNA sequence of mouse *Tfdp1* was cloned into a pLVX‐puro lentiviral vector. Lentivirus was produced in 293T cells following standard protocols. Activated CD4^+^ T cells were infected with TFDP1‐overexpressing lentivirus (or empty vector control) in the presence of 8 µg/mL polybrene and 2 µg/mL puromycin for 48 hours. After infection, cells were harvested, washed, and resuspended in sterile PBS for in vivo injections or in vitro assays.

### 4.23 DSS Model With CD4^+^ T Cell Reconstitution

Mice were administered 2.5% DSS in drinking water starting on day 1 and received an intraperitoneal injection of 200 µg anti‐CD4 monoclonal antibody (CD4Ab) on the same day, followed by 100 µg CD4Ab every 3 days thereafter. On day 4, 2 × 10^6^ modified CD4^+^ T cells (WT, *Ddx21*–deficient, or *Ddx21*–deficient overexpressing *Tfdp1*) were intravenously injected, and CD4Ab administration was discontinued (clone GK1.5, Leinco Technologies, Inc.) [52]. On day 5, DSS was withdrawn and replaced with normal drinking water. Mice were euthanized on day 8 for analysis.

### 4.24 CD8^+^ T Cell Depletion In Vivo

To investigate the contribution of CD8^+^ T cells to DSS‐induced colitis, CD8^+^ T cells were depleted in vivo using a specific anti‐CD8α monoclonal antibody. *Ddx21*‐cKO mice and their WT littermate controls were treated with 2.5% DSS in drinking water for 4 consecutive days, followed by 4 days of normal drinking water. On day 1 and day 4 of the experimental period, mice received intraperitoneal injections of 15 µg per mouse of an anti‐CD8α depleting antibody (clone 53–5.8, Bio X Cell). Control mice were injected with an equal volume of isotype‐matched control antibody. The efficiency of CD8^+^ T cell depletion was confirmed by flow cytometric analysis of splenocytes at the end of the experiment.

### 4.25 Statistics

Unpaired Student's *t‐*tests, one‐way analysis of variance (ANOVA), and two‐way ANOVA were used to compare pairs of groups, and all data are presented as the means ± SEM. *p* < 0.05 was considered statistically significant. Statistical analyses were performed using Prism (GraphPad 9.4.0). Heatmap, Volcano plot, and Venn diagram were drawn using the OmicStudio tool on https://www.omicstudio.cn/tool.

## Author Contributions

Y.Z. was responsible for research design, conducting experiments, analyzing the data, and preparing the initial draft of the manuscript. C.K. and Y.H. performed experiments and analyzed data. X.Y. performed the bioinformatic analysis. X.C., Q.Z., and Y.M. performed experiments. Z.W. contributed to the discussion. N.J. collected the tissue samples. L.W. provided valuable discussions and suggestions throughout the project. H.‐B.L. originated the project idea, oversaw the study design, directed the research activities, and was involved in both writing and revising the manuscript. J.Z. contributed to research coordination, experiment execution, and manuscript preparation and revision.

## Conflicts of Interest

The authors declare no conflicts of interest.

## Supporting information




**Supporting File**: advs75836‐sup‐0001‐SuppMat.docx.

## Data Availability

All data needed to evaluate the conclusions in the article are present in the article and/or the Supplementary materials. The datasets used and/or analyzed during the current study are available from the corresponding author upon reasonable request. The RNA sequencing data were deposited in the BioProject database under BioProject ID: PRJNA1244719 (https://www.ncbi.nlm.nih.gov/sra/PRJNA1244719). The mass spectrometry proteomics data have been deposited to the ProteomeXchange Consortium (https://proteomecentral.proteomexchange.org) via the iProX partner repository [70, 71] with the dataset identifier PXD062495.
