# Supplementary material for: RNA Helicase DDX21 Controls CD4+ T Cell Proliferation and Promotes Inflammatory Bowel Disease via Translational Control
Source: Adv Sci (Weinh). 2026 May 29;13(47):e16653. doi: 10.1002/advs.202516653 (PMC13336107; doi:10.1002/advs.202516653)
Supplement: Supplementary file 1 [file ADVS-13-e16653-s001.docx]

**Supplementary figure legends:**

**Fig S1. RNA and protein levels of DDX21 during CD4^+^ T cells activation.**

**a** DDX21 protein expression was analyzed using a previously published proteomics dataset (PRIDE accession: PXD004367)^[8]^. **b** *Ddx21* mRNA expression was analyzed using our previously published RNA-seq dataset (GEO accession: GSE184909)^[29]^. **c** DDX21 protein levels was analyzed by Western blot in naïve CD4^+^ T cells activated with anti-CD3/CD28 antibodies for the indicated time points. **d** *Ddx21* mRNA expression was analyzed by real-time PCR in naïve CD4^+^ T cells activated with anti-CD3/CD28 antibodies for the indicated time points; results were analyzed by one-way ANOVA (*n* = 3). Data are shown as the means ± SEM. ****p* < 0.001, *****p* < 0.0001.

**Fig S2. Flow cytometry gating strategy for thymocyte and T cell subsets.**

**a** Flow cytometry gating strategy for thymocyte. **b** Flow cytometry gating strategy for T cell subsets and Ki67 expression in the spleen.

**Fig S3. *Ddx21*-deficiency did not affect T cell development in the thymus.**

**a** Strategy for targeting the 3-5 introns of *Ddx21* gene to construct *Ddx21*^flox/flox^ mice. **b** Protein level of DDX21 was analyzed by Western blot in splenic CD4^+^ T cells isolated from *Ddx21*-cKO and WT control mice, with β-actin used as the internal control. **c** Representative dot plots displaying the composition of CD4^+^ T and CD8^+^ T cells in the thymus of *Ddx21*-cKO and WT littermate mice. **d-e** Ratios (**d**) and absolute numbers (**e**) of different T cell subsets in the thymus are shown (*n* = 3-6); results were analyzed by unpaired *t*-test. Data in **b-e** represent one of three independent experiments and are shown as the means ± SEM, **p* < 0.05. NS, not significant.

**Fig S4. T cell-specific *Ddx21* deletion disrupts peripheral CD8^+^ T cell homeostasis.**

**a** Representative dot plots show CD8^+^ T cell composition, as well as the expression of CD44 and CD62L on CD8^+^ T cells from *Ddx21*-cKO and WT littermate mice at a steady state in the spleen. **b-c** Ratios (**b**) and absolute numbers (**c**) of CD8^+^ T cells in the spleen are shown. **d** Ratios of CD44^lo^ CD62L^hi^ and CD44^hi^ CD62L^lo^ cells in splenic CD8^+^ T cells are shown. **e** Representative dot plots showing apoptosis in splenic CD8^+^ T cells. **f** Percentage of apoptotic CD8^+^ T cells in the spleen. **g** Percentage of Ki67^+^ CD8^+^ T cells in the spleen. **h** Percentage of IFN-γ^+^, TNF-α^+^, and Granzyme B^+^ cells among CD8^+^ T cells in the spleen of *Ddx21*-cKO mice and WT littermates. Data represent one of three independent experiments and are shown as the means ± SEM, *n* = 3-5 biologically independent samples. **p* < 0.05. ***p* < 0.01, ****p* < 0.001, *****p* < 0.0001. NS, not significant; results were analyzed by unpaired *t*-test.

**Fig S5. Gut microbial profiles of co-housed *Ddx21*-cKO and WT mice.**

**a** Principal Coordinates Analysis (PCoA) of gut microbiota in *Ddx21*-cKO mice and WT littermates (*n* = 5). **b** Relative abundance distribution of species at the phylum level across groups.

**Fig S6. *Ddx21* deficiency alleviates DSS-driven colitis.**

**a-b** DDX21 expression in macrophages **(a)** and dendritic cells **(b)** was evaluated by mean fluorescence intensity (MFI). CD45^+^ MHC-II^+^ F4/80^+^ CD11b^+^ cells were gated as macrophage, and CD45^+^ MHC-II^+^ F4/80^-^ CD11b^+^ CD11c^+^ cells were gated as dendritic cell (*n* = 3). Results were analyzed by an unpaired *t*-test. **c** Body weight changes during colitis development in *Ddx21*-cKO and WT mice treated with 2.5% DSS for 4 days, followed by 4 days of normal drinking water; results were analyzed by two-way ANOVA (*n* = 3). **d**-**e** Representative images of mouse colons (**d**) and colon length measurements (**e**) on day 8 in **c** (*n =* 3); results were analyzed by an unpaired *t*-test. **f** The DAI of mice were analyzed by two-way ANOVA (*n* = 3). **g** Representative images of H&E staining of the colons on day 8 (*n =* 3); scale bar=100 μm. The percentages of CD4^+^ T cells (**h**) and Ki67^+^ CD4^+^ T cells (**i**) in **c** at day 8 from colon of *Ddx21*-cKO mice and WT littermates were determined (*n =* 3); results were analyzed by an unpaired *t*-test. **j** Percentage of IFN-γ^+^, IL-10^+^, and IL-17A^+^ cells among CD4^+^ T cells in the colon of *Ddx21*-cKO mice and WT littermates on day 8 (*n* = 3); results were analyzed by an unpaired *t*-test. Data represent one of three independent experiments and are shown as the means ± SEM, **p* < 0.05. ****p* < 0.001, *****p* < 0.0001. NS, not significant.

**Fig S7. Differentiation of *Ddx21-*deficient CD4^+^** **T cells *in vitro*.**

**a**. Naïve CD4^+^ T cells were differentiated into Th1 subset under defined optimal conditions. The percentage is listed in the lower graph (*n* = 3); results were analyzed by unpaired *t*-test. **b**. Naïve CD4^+^ T cells were differentiated into Th2 subset under defined optimal conditions. The percentage is listed in the lower graph (*n* = 3); results were analyzed by unpaired *t*-test. **c.** Naïve CD4^+^ T cells were differentiated into Th17 subset under defined optimal conditions. The percentage is listed in the lower graph (*n* = 3); results were analyzed by unpaired *t*-test. **d**. Naïve CD4^+^ T cells were differentiated into iTreg subset under defined optimal conditions. The percentage is listed in the lower graph (*n* = 3); results were analyzed by unpaired *t*-test. Data represent one of three independent experiments and are shown as the means ± SEM. **p* < 0.05, ***p* < 0.01, ****p* < 0.001, *****p* < 0.0001.

**Fig S8. Effects of CD8^+^ T cells on T cell-driven colitis.**

**a** Body weight changes were monitored in *Ddx21*-cKO and WT mice during colitis development. Mice were treated with 2.5% DSS in drinking water for 4 days, followed by 4 days of normal drinking water. On days 1 and 4, mice received intraperitoneal injections of 15 μg/mouse of CD8 depletion reagent or IgG control depletion reagent (*n =* 3); results were analyzed by two-way ANOVA. **b**-**c** Representative images of mouse colons (**b**) and colon length measurements (**c**) on day 8 in **a** (*n =* 3); results were analyzed by one-way ANOVA. **d** The DAI of mice were analyzed by two-way ANOVA (*n*  = 3). **e** Representative images of H&E staining of the colons on day 8 (*n =* 3); scale bar=100 μm. Data represent one of three independent experiments and are shown as the means ± SEM, **p* < 0.05. ***p* < 0.01, ****p* < 0.001, *****p* < 0.0001.

**Fig S9. DDX21 deletion in T cells protects against experimental autoimmune encephalomyelitis.**

**a** *Ddx21*-cKO mice and WT littermates were immunized with the MOG_35–55_ peptide emulsified in a complete Freund’s adjuvant. Daily clinical experimental autoimmune encephalomyelitis (EAE) scores are shown (*n* = 3); results were analyzed by two-way ANOVA. **b** Representative dot plots show the composition of CD4^+^ T cells and CD8^+^ T cells in the CNS of *Ddx21*-cKO mice and WT littermates following EAE induction. **c** Percentages and cell number of CD4^+^ T cells and CD8^+^ T cells in (**b**) are shown (*n* = 3); results were analyzed by unpaired *t*-test. Data represent one of three independent experiments and are shown as the means ± SEM. **p* < 0.05, ***p* < 0.01, *****p* < 0.0001. NS, not significant.

**Fig S10. DDX21 regulates key transcription factors for cell cycle and DNA replication**

**a** Scatter plot with a Spearman correlation coefficient indicates the relationship between *DDX21* and *RPS20* expression in CD4^+^ T cells, utilizing data from normal and inflamed tissues across various inflammatory bowel disease (IBD) datasets (Merged: combining the used datasets together; Smillie *et al*. (Single Cell Portal: SCP25922^[23]^); Kong *et al*. (Single Cell Portal: SCP188423^[24]^); Zilbauer et al. (Biostudies: E-MTAB-890124^[25]^); and Zhao et al. (GEO accession: GSE24208725^[26]^)). **b** Direct interaction between DDX21 and *Tfdp1* analyzed via RIP-qPCR (*n* = 3); results were analyzed by an unpaired *t*-test. **c** The dot plot displays the expression of *DDX21* in proliferating T cells and conventional CD4^+^ T cells from scRNA-seq datasets (Acute Lymphocytic Leukemia (ALL, GSE132509), Chronic Lymphocytic Leukemia (CLL, CLL-1: GSE152469, CLL-2: GSE111014). The size of the dots corresponds to the expression levels of *DDX21*, with larger dots indicating a higher expression. **d-e** Detection of *DDX21* and *TFDP1* mRNA (**d)** and protein expression (**e)** in Jurkat cells following DDX21 knockdown via real-time PCR and Western blot analysis (*n* = 3); results were analyzed by an unpaired *t*-test. **f** Apoptosis of Jurkat cells with or without DDX21 was analyzed using the PE Annexin V Apoptosis Detection Kit with 7-AAD (BioLegend). Representative dot plots are shown. **g** Ratios of Annexin V^+^ 7-AAD^-^ and Annexin V^+^ 7-AAD^+^ cells in **f** are shown (*n* = 3). **h-i** Detection of *Tfdp1* mRNA expression (**h**) and protein expression (**i**) in *Ddx21*-deficient and wild-type (WT) CD4^+^ T cells following anti-CD3/CD28 antibody stimulation, as well as in *Ddx21*-deficient T cells overexpressing TFDP1 (*n* = 3); results were analyzed by one-way ANOVA. Fluorescent images of DDX21 (**j**), RPS20 (**k**), TFDP1 (**l**) and CD4 staining combined with DAPI counterstaining in normal and inflammatory tissues from IBD patients; scale bar = 20 µm. Data represent one of three independent experiments and are shown as the means ± SEM, **p* < 0.05, ***p* < 0.01, ****p* < 0.001, *****p* < 0.0001. NS, not significant.

**Fig S11. TFDP1 overexpression restores the pathogenic capacity of *Ddx21*-deficient CD4^+^ T cells *in vivo.***

**a-g** Mice were administered 2.5% DSS in drinking water starting on day 1 and received an intraperitoneal injection of 200 μg anti-CD4 monoclonal antibody (CD4Ab) on the same day, followed by 100 μg CD4Ab every three days thereafter. In the CD4^+^ T cell reconstitution groups, 2 × 10^6^ CD4^+^ T cells (WT, *Ddx21*‑deficient, or *Ddx21*‑deficient overexpressing *Tfdp1*) were intravenously injected on day 4, at which point CD4Ab administration was discontinued. On day 5, DSS was withdrawn and replaced with normal drinking water. Mouse body weights were recorded daily (*n* = 5) (**a**); results were analyzed by two‑way ANOVA. Representative images of mouse colons (**b**) and colon length measurements (**c**) on day 8 (*n* = 5); results were analyzed by one‑way ANOVA. Disease activity index (DAI) scores were assessed daily (**d**); results were analyzed by two‑way ANOVA (*n* = 5). Representative images of H&E‑stained colon sections on day 8 (**e**); scale bar = 100 μm (*n* = 5). Flow cytometric analysis of transferred CD4^+^ T cells in the colon on day 8 (**f**) and the percentages of colonic CD4^+^ T cells (**g**) are shown (*n* = 5); results were analyzed by one‑way ANOVA. **h-n** 5 × 10^5^ CD4^+^ T cells (WT, *Ddx21*‑deficient, or *Ddx21*‑deficient overexpressing *Tfdp1*) were adoptively transferred into *Rag1*^−/−^ mice. Body weights of recipient mice were measured weekly (**h**); results were analyzed by two‑way ANOVA (*n* = 5). Nine weeks after transfer, mice were euthanized; representative images of mouse colons (**i**) and colon length measurements (**j**) were obtained (*n* = 5); results were analyzed by one‑way ANOVA. DAI scores were assessed weekly (**k**); results were analyzed by two‑way ANOVA (*n* = 5). Representative images of H&E staining of colonic sections from recipient mice on week 9 are shown (**l**); scale bar = 100 μm (*n* = 5). Flow cytometric analysis of transferred CD4^+^ T cell populations in the colon on week 9 (**m**) and the percentages of colonic CD4^+^ T cells (**n**) are presented (*n* = 5); results were analyzed by one‑way ANOVA. Data represent one of two independent experiments and are shown as the means ± SEM. **p* < 0.05, ***p* < 0.01, ****p* < 0.001, *****p* < 0.0001.

**Fig S12. Pharmacological inhibition of ribosomal biogenesis suppresses CD4^+^ T cell viability.**

**a-b** Expression levels of ATM (**a**) and phosphorylated ATM (p-ATM) (**b**) in *Ddx21*-deficient CD4^+^ T cells, WT CD4^+^ T cells, and WT CD4^+^ T cells treated with 10 μM KU55933 for 24 hours, assessed by mean fluorescence intensity (MFI) (*n* = 3); results were analyzed by one-way ANOVA. **c** The effects of 24-hour KU55933 treatment on the viability of CD4^+^ T cells, macrophages, and epithelial cells were assessed using the CCK-8 assay kit (*n* = 3); results were analyzed by unpaired *t*-test. **d** Flow cytometry analysis of protein synthesis of activated CD4^+^ T cells (treated with or without 10 μM KU55933), assessed by the mean fluorescence intensity (MFI) of O-propargyl-puromycin (*n* = 3); results were analyzed by unpaired *t*-test. **e** Flow cytometry analysis of the cell cycle of activated CD4^+^ T cells (treated with or without 10 μM KU55933) is shown; results were analyzed by unpaired *t*-test. Data represent one of three independent experiments and are shown as the means ± SEM. **p* < 0.05, ***p* < 0.01, ****p* < 0.001, *****p* < 0.0001. NS, not significant.

**Fig S13. Safety evaluation of KU55933 *in vivo.***

**a** Mice were administered different doses of KU55933 twice weekly; body weight was monitored every two days, and results were analyzed by two-way ANOVA (*n* = 3). **b-c** Representative colon images **(b)** and length **(c)** measurements in **a** at day 8 (*n* = 3); results were analyzed by one-way ANOVA. **d** Organ-to-body weight ratios of the liver, kidney, and spleen on day 8 (*n* = 3); results were analyzed by one-way ANOVA. **e** Representative images of H&E staining of colon, liver, kidney, and spleen on day 8; scale bar=100 μm. **f** Serum biochemical parameters (Cr, BUN, AST, ALT) were measured on day 8 using commercially available kits (*n* = 3); results were analyzed by one-way ANOVA. Data represent one of three independent experiments and are shown as the means ± SEM.

**Fig S14. Proposed model of DDX21-mediated translational regulation during T cell expansion.** Upon T cell activation, DDX21 promotes ribosome biogenesis, which is essential for efficient translation of *Tfdp1* mRNA. The resulting TFDP1 protein drives cell cycle progression, enabling robust T cell expansion required for immune responses. DDX21 deficiency disrupts this TFDP1 translational regulatory axis, ultimately impairing T cell proliferation.

Fig.S1

Fig.S2

Fig.S3

Fig.S4

Fig.S5

Fig.S6

Fig.S7

Fig.S8

Fig.S9

Fig.S10

Fig.S11

Fig.S12

Fig.S13

Fig.S14

| **Supplementary Table 1.** catRAPID prediction results for the binding between DDX21 and *Tfdp1* mRNA | | | | | |
| --- | --- | --- | --- | --- | --- |
| rnaFrag_start | rnaFrag_end | Interaction_Propensity | Z_score | RNA_Binding_Domains_IDs | Ranking |
| 958 | 1059 | 74.78 | 2.69 | PF00270,PF08152,PF00271,PF04851 | 0.612083 |
| 951 | 1052 | 68.98 | 2.37 | PF00270,PF08152,PF00271,PF04851 | 0.598625 |
| 1608 | 1709 | 53.87 | 1.53 | PF00270,PF08152,PF00271,PF04851 | 0.563625 |
| 1601 | 1702 | 48.56 | 1.23 | PF00270,PF08152,PF00271,PF04851 | 0.551333 |
| 1158 | 1259 | 40.62 | 0.79 | PF00270,PF08152,PF00271,PF04851 | 0.532958 |
| 651 | 752 | 39.02 | 0.7 | PF00270,PF08152,PF00271,PF04851 | 0.52925 |
| 358 | 459 | 36.82 | 0.58 | PF00270,PF08152,PF00271,PF04851 | 0.524125 |
| 1451 | 1552 | 34.59 | 0.46 | PF00270,PF08152,PF00271,PF04851 | 0.518958 |
| 351 | 452 | 32.9 | 0.36 | PF00270,PF08152,PF00271,PF04851 | 0.515083 |
| 258 | 359 | 32.82 | 0.36 | PF00270,PF08152,PF00271,PF04851 | 0.514875 |
| 1151 | 1252 | 27.28 | 0.05 | PF00270,PF08152,PF00271,PF04851 | 0.502042 |
| 958 | 1059 | 74.78 | 2.69 | PF00270,PF08152,PF00271,PF04851 | 0.612083 |
| 951 | 1052 | 68.98 | 2.37 | PF00270,PF08152,PF00271,PF04851 | 0.598625 |
